# Academic integrity across educational levels: Exploring students’ engagement with grey-zone and non-compliant practices in four European countries

**DOI:** 10.1371/journal.pone.0342227

**Published:** 2026-03-04

**Authors:** Mikkel Willum Johansen, Mads Paludan Goddiksen, Christine Clavien, Linda Hogan, I. Anna S. Olsson, Júlio Borlido Santos, Rita Alves dos Santos, P.J. Wall, Peter Sandøe, Thomas Bøker Lund

**Affiliations:** 1 Department of Science Education, University of Copenhagen, Copenhagen, Denmark; 2 Department of Food and Resource Economics, University of Copenhagen, Denmark; 3 Institute for Ethics, History and the Humanities, University of Geneva, Switzerland; 4 School of Ecumenics, Trinity College Dublin, Ireland; 5 i3S – Instituto de Investigação e Inovação em Saúde, Universidade do Porto, Portugal; 6 Vrije Universiteit, Amsterdam, Netherlands; 7 ADAPT Centre, School of Computer Science and Statistics, Trinity College Dublin, Ireland; 8 Department of Veterinary and Animal Sciences, University of Copenhagen, Denmark; University of Southampton, MALAYSIA

## Abstract

To foster academic integrity in students and future scholars, it is essential to understand how their integrity behaviours evolve throughout their educational trajectory and across various academic integrity topics. While much research has examined students’ perception of and engagement in plagiarism and other forms of clear-cut cheating, grey-zone practices have largely been neglected, and comparisons across educational levels are rare. This paper presents a comprehensive overview of European students’ conceptions of and engagement with less clearcut aspects of academic integrity, and the potential effects of academic integrity training. The study draws on a large-scale survey of 3,297 students from Denmark, Ireland, Portugal, and Switzerland, covering three educational levels (upper secondary, Bachelor, and PhD). The survey examined perceptions of and engagement in likely grey-zone and non-compliant practices across three dimensions of academic integrity: i) Plagiarism and citation practice, ii) Collaborative practices, and iii) Data collection and analysis. Responses were analysed using descriptive statistics and regression analyses. Results showed that participants at higher educational levels were better at identifying likely non-compliant practices related to plagiarism and citation, and they were less likely to have engaged in such practices during their current studies. Progress along the educational trajectory was less pronounced regarding collaborative practices and practices related to data collection and analysis. In particular, 14% of the PhD level participants admitted having deleted deviating data “based on a gut feeling that they were inaccurate” and 20% admitted to keeping inaccurate records. All participants had a low level of competence in identifying grey-zone practices, and strikingly, their competences did not improve along their educational trajectory. Academic integrity training was not consistently correlated with any group of participants’ competences regarding likely grey-zone practices, although it was positively correlated with upper secondary and PhD participants’ competences concerning certain likely non-compliant practices. These results call for a different approach to academic integrity training. In particular, they call for more comprehensive approaches that include grey-zone as well as non-compliant practices, and address a broad range of questionable behaviours, not only plagiarism.

## 1. Introduction

Over the past decades, a number of key areas of focus have emerged in the study of academic integrity among students in higher education. For upper secondary and undergraduate university students, different types of cheating – especially plagiarism – have been a major area of focus [e.g., 1–9]. These studies show that cheating – and plagiarism in particular – is common among upper secondary students. In an early study, Schab [1] found that 76% of a population of American upper secondary students admitted to having plagiarised, and 68% admitted to using cheat sheets. Roughly comparable levels of transgressions were also found in later studies and studies from other countries [e.g., 6, 10–12], with Taiwanese students as the exception, reporting markedly lower levels (<3%) of plagiarism [7]. The level of exam-based cheating among undergraduate university students is reported to be somewhat lower. Based on a survey of more than 64,000 American and Canadian undergraduate college students, McCabe [3] reported that around 21% admitted to committing some form of severe exam-related cheating, such as copying from another student, using crib notes, or helping others to cheat. Hopp & Speil [9] found that more than 20% of a population of Austrian college students admitted to plagiarising. As an outlier, Curtis and Tremayne [8] observed that about 64% of a population of undergraduate students from Western Sydney University admitted to having engaged in some form of plagiarism at least once. The study did, however, also find that levels of cheating were on the decline compared with earlier studies of the same population.

In addition to investigations of the prevalence of cheating, students’ motivation to, perception of, and attitude towards cheating have also been areas of focus [e.g., 3, 12–23]. As an example, McCabe [3] investigated students’ and faculty members’ perception of the seriousness of a selection of different forms of cheating, such as copying from other students or using crib notes. Results showed that fewer students considered these behaviours to be moderate or serious cheating compared to faculty members. A slightly different approach was taken by Roig [13], where participants were given different paraphrases of a short text and for each paraphrase asked to determine whether it complied with regulations. Results showed that 40–50% of the participants believed some of the non-compliant paraphrases were acceptable.

Other types of problematic behaviour, such as outsourcing assessments to third parties (known as ‘contract cheating’), fraud, and corruption (e.g., bribing teachers) have also been given some attention [e.g., [24–26]].

Questionable authorship practices have been an important additional area of focus for PhD level students. To mention two recent studies, Helgesson and colleagues [27] reported that 46% of a population of medical PhD students said that the standard ICMJE authorship criteria had not been fully respected in at least one of the papers included in their dissertation. Similarly, Goddiksen and colleagues [28] found that 34% of a cross-faculty population of PhD students in Europe believed that they had granted at least one undeserved authorship to a person in power during their PhD studies.

Questions about falsification and fabrication of data have received relatively little attention in the literature on student integrity (see [29] for a meta-analysis). One exception is Johansen & Christiansen [30], who found that 64% of a population of Bachelor students in chemistry programmes at Danish universities had deleted outliers or discarded experiments only because the results seemed wrong or because they felt that there was a fault in the measurement. Furthermore, the study showed that large groups of students had been encouraged by a teacher to delete anomalous data.

As a final theme, various approaches to academic integrity training have been reported. McCabe and Treviño [31] found the introduction of honour codes to be an effective tool in reducing cheating among university and college students. Later research, however, revealed that honour codes are only effective in reducing cheating if they are part of a larger culture supporting integrity [5:106, 32]. This result resonates well with a large body of studies showing contextual factors such as culture and the perception of peer behaviour to be major explanatory factors in students’ integrity behaviour and attitudes [e.g., 33, 34].

Another line of research has sought to develop and evaluate specific educational interventions, but with mixed results. Some studies report a clear effect of simple interventions such as online courses [e.g., 35], while others show a limited effect [e.g., 36]. In one of few effect-control group studies, Goddiksen and colleagues [37] compared a traditional text-based training module with training utilising an interactive online platform. Both interventions significantly improved participants’ understanding of academic integrity, but contrary to expectations, no difference between the effect of the two modes of training could be observed.

In a meta-analysis of 30 studies reporting the effect of training interventions, Katsarov and colleagues [38] demonstrated that interventions are most effective if they allow for a constructive approach where learners are taught to apply guidelines to complex cases. As an important limitation to this line of research, a meta-analysis Stoesz & Yudintseva [39] found that few of the effect studies investigated whether the evaluated intervention led to long-term improvement in student behaviour.

As the short review above shows, the literature on academic integrity in higher education is comprehensive and addresses several key issues, primarily: prevalence of cheating, attitudes towards it, integrity training approaches, and questionable authorship practices among PhD students. However, certain important areas remain underexplored. For example, the vast majority of the studies focus only on a single educational level. This makes it difficult to systematically map and compare students’ behaviour and understanding of academic integrity across levels. If we want to improve the academic integrity of students and future academics, it is imperative to know how their integrity behaviour changes throughout their educational trajectory, especially during the formative years from upper secondary to graduate school. Such an overview can provide valuable background knowledge when planning interventions at specific levels. It may also help to identify gaps and topics for which students are likely to lack the training they will need when progressing to the next educational level.

Furthermore, as discussed by Goddiksen and colleagues [23], most of the current academic integrity literature focuses on practices that are (at least from the teachers’ point of view) clearly non-compliant. While it is important that students and academics know the rules and regulations well enough to handle such clear-cut situations correctly, these do not exhaust the landscape of academic integrity. Many of the integrity issues students encounter in their daily lives fall within a ‘grey zone’, where simply knowing the rules is not enough to handle the issues ethically. In these cases, it is also necessary to consider the context and to be aware that there may not always be a single correct answer to an ethical dilemma [cf. 23, 30, 40].

In this study, we will address these gaps in the literature by reporting from a large-scale survey of upper secondary, Bachelor, and PhD students in Europe. The main aims of the study are: 1) to provide a systematic and extensive overview of European students’ conceptions of and engagement with academic integrity and 2) to analyse the relationship between students’ integrity-related behaviour and academic integrity training as well as other demographic background variables (gender, age, country of study).

The study aims to be comprehensive in terms of both educational levels and types of integrity issues covered. Regarding educational levels, the study includes upper secondary, Bachelor, and PhD students, thus enabling systematic comparisons across these three levels. Regarding types of integrity issues, the study addresses likely grey-zone as well as likely non-compliant actions, and it covers three dimensions of academic integrity in roughly equal measures: i) plagiarism and citation practice, ii) collaborative practices, and iii) data collection and analysis.

The comprehensive overview and analysis of students’ perception and behaviour and the relationship with academic integrity training will allow us to identify possible blind spots and areas of potential improvement in the current approaches to academic integrity for upper secondary, university, and PhD students in Europe.

## 2. Materials and methods

The study is based on a subset of data from a questionnaire-based survey undertaken as part of the project INTEGRITY (https://h2020integrity.eu/). The survey aimed to map academic integrity within the European Economic Area (EEA) across educational levels from upper secondary to PhD, and across different fields of study. Only data from questions concerning demographic background, academic integrity training, competences and conceptions of academic practices, and own questionable practices are included in this study.

Four studies based on parts of the survey data have previously been published [23, 28, 40, 41]. These studies explore Bachelor students’ conceptions of academic integrity [23], upper secondary students’ conceptions of and academic integrity behaviour [40], PhD student’s experiences with authorship attribution [28], and upper secondary and Bachelor student’s experiences with text matching software [41]. This study adds to these by presenting and analysing data concerning own behaviour from Bachelor and PhD students and conception of rules from PhD students not previously analysed. Furthermore, in this study, results and analyses are compared across educational levels to reach the first research objective. No further manuscripts based on the survey data are planned, in press or in review.

### 2.1. Ethics

The study was reviewed and approved by the Research Ethics Committee for Science and Health at the University of Copenhagen prior to the pilot tests (ref. no. 504–0043/18–5000). Participation in the study was voluntary and anonymous. Participants were not compensated for participating. For participants above the age of 18, written informed consent to participate was obtained via the first question in the questionnaire (see S1 File). For participants below the age of 18, parental consent was also collected and retained by the participating institutions. Additional information regarding the ethical, cultural, and scientific considerations specific to inclusivity in global research is included in the Supporting Information S5 File.

### 2.2. Participants and data collection

Data were collected in nine EEA countries or areas, using an anonymous online survey from January 2020 to December 2020. The overall project aimed to have at least 200 participants from each of the study levels. To achieve this, different recruitment strategies were used for the different study levels.

For the upper secondary level, we used an institution-level sampling design. A complete list of upper secondary institutions was compiled for each country, and institutions were randomly drawn from the list and invited to participate until a target of approximately 200 participants was met (see Johansen and colleagues [40] for further details).

For Bachelor level, we used a programme-level sampling design. For each country, a complete list of Bachelor programmes was compiled, and programmes were randomly drawn from the list and invited to participate. In addition to the target of approximately 200 participants, we also aimed to include at least 45 participants from each of the three scholarly fields: humanities, social sciences, and STEMM (Science, Technology, Engineering, Mathematics, and Medicine). Only students who had completed at least one year of their studies (60 ECTS) were invited to complete the survey (see Goddiksen and colleagues [23] for further details).

For PhD level, the recruitment strategy differed from country to country due to large variation in institutional organisation, as well as the number of PhD students. In addition to the target of approximately 200 participants, we aimed to include at least 45 participants from each field (humanities, social sciences, and STEMM). For countries and faculties with few potential participants, total population recruitment was carried out, while a programme-level sampling design was used in countries and faculties with many potential participants (see [28] for further details).

For the analysis in this paper, we included countries with at least 200 participants from each study level. This target was reached for Denmark, Ireland, Portugal, and the French-speaking part of Switzerland, but not the German-speaking part of Switzerland, Germany, Hungary, Lithuania, the Netherlands, or Slovenia (Table 1). Consequently, the final dataset for this study consists of 3,297 participants from the four EEA countries or areas shown as non-shaded in Table 1. Note that we only reached 199 Bachelor level participants in the French-speaking part of Switzerland, but we considered this number to be sufficiently close to the target to include the area in the study. See [23,28,40] for detailed information about each of the three populations.

**Table 1:** Overview of all survey participants by country and study level. Shaded countries were not included in the sample for this study due to insufficient data for one or more study levels.

Country	Upper secondary	Bachelor	PhD	Total
Denmark	389	218	427	1,034
Germany	17	94	85	196
Hungary	60	292	221	573
Ireland	292	231	245	768
Lithuania	215	204	64	483
The Netherlands	30	96	171	297
Portugal	219	274	241	734
Switzerland*	360	199	202	761
Switzerland**	76	11	59	146
Slovenia	250	221	48	519
Total	1,908	1,840	1,763	5,511
**Total in sample** (non-shaded area)	1,260	922	1,115	3,297

* French-speaking part of Switzerland

** German-speaking part of Switzerland

### 2.3. Materials and measures

The questionnaire was developed using a qualitative interview study (see [23,42] for details). The questionnaire was pilot tested, translated into the dominant language of each of the nine participating countries and implemented as an anonymous online survey using the platform SurveyXact ver. 12.9 (https://www.surveyxact.com/). The pilot testing involved both qualitative and quantitative tests of the questionnaire. The translation was carried out following a ‘translation back-translation’ protocol (c.f. [40]).

The questionnaire was adaptive, such that the questions presented to a particular participant would depend on their educational level and background. For Bachelor and PhD students, only the participants who stated that they worked with data were presented with all the questions relating to data, and the wording of the questions was designed to fit the type of data they primarily used (qualitative, quantitative, historical sources, or works of art). Similarly, the wording of the questions was designed to fit the educational level of the participants. See S1 File for details. Here, we focus on the parts of the survey reported in this study. These can be divided into four main categories.

1 **Demographics.** These questions included standard demographic details (age, gender identity, country of study) and study-specific details (name of educational institution, educational level, and for Bachelor and PhD students, their general area of study and the type of data they primarily used).
2 **Competences and conceptions of academic practices.** Participants’ competence in relation to academic integrity was explored through two different approaches: a) a direct test of competences in relation to the use of others’ texts and b) a survey of participants’ conceptions of rules of academic practice and how these rules apply to specific situations.

In the direct test of competences, we used a scenario-based approach inspired by Roig [13]. Participants were presented with a short text (41 words) and were told that a friend wanted to use it in an assignment/paper they were currently writing. Participants were then shown four different paraphrases of the text, and for each one they were asked whether their friend had acted in an acceptable way by using the paraphrase in question. The four paraphrases can be described as follows (see S1 File for details):

- Paraphrase 1: A direct copy with no citation marks and no reference to the original.
- Paraphrase 2: Some insignificant words had been changed to synonyms. There was no reference to the original.
- Paraphrase 3: Same as paraphrase 2 but with a reference to the original.
- Paraphrase 4: A more substantial rewriting with a reference to the original.

To assess the participants’ conceptions of rules, they were presented with descriptions of 11 academic practices: four relating to citation and plagiarism, four to collaboration, and three to data collection (Table 2). For each academic practice, participants were asked if they believed the practice was “against the rules and regulations that apply to you”. Participants could answer by choosing one of the options: “Yes, it is a serious violation”, “Yes, but it is not a serious violation”, “No, it is not against the rules”, “The rules are unclear”, “It depends on the situation”, and “I don’t know”. The first three of these options indicate a belief that the rules and regulations apply to the practice. The fourth and fifth option, on the other hand, indicate the belief that the rules do not readily apply to the practice.

**Table 2:** List of academic practices used to assess the participants’ conceptions of rules. The practices are categorised as likely non-compliant, or as likely grey-zone practices if their evaluation depends significantly on contextual information. The exact wording in the description of the practices depended on the educational level and type of data used by the participants. Here we have used the wording presented to upper secondary and Bachelor level participants using quantitative data (see S1 File for details about the wording for all study levels and data uses).

	Likely non-compliant	Likely grey-zone
Citation practices		
Copying an entire page stating a central point from an external source into your own text without quotation marks but including a reference.	X	
Copying one short paragraph stating a central point from an external source into your own text without quotation marks but including a reference.	X	
Changing 10% of the words in a short paragraph stating a central point from an external source and using it in your own text with a reference.		X
Copying a central point formulated in half a sentence from an external source without marking it with quotation marks but including a reference.		X
Collaborative practices*		
Paying someone to write an assignment for you.	X	
Comparing answers to an individual assignment with other students before handing in the assignment.		X
Handing in an assignment that you made with extensive help from another student or family member, without mentioning the help you received.		X
Letting one member of a group do all the writing on a group project while the other members contribute to the analysis and literature search.		X
Data collection		
Not mentioning in an assignment that you replaced a number of outliers in a dataset with data points obtained through estimates based on the remaining data points.	X	
Not mentioning in an assignment that you removed a number of deviating data points from a dataset when the cause of the deviation was unknown.	X	
Not mentioning in an assignment that you removed a number of deviating data points from a dataset when the cause of the deviation was known.		X

* PhD students were not asked this set of questions.

The academic practices were constructed such that half were likely non-compliant, while the other half were likely ‘grey-zone’ practices in the sense that an evaluation of the practice would depend on contextual information. For instance, paying someone to write an assignment for you is likely non-compliant, whereas it is likely to depend on the context and precise instruction whether or not it is against the rules to let one member of a group do all the writing on a group project while the other members contribute to the analysis and literature search. Table 2 presents the full list of academic practices and our categorisation of these (see also [23] for elaboration).

3 **Academic integrity training.** The questionnaire included two questions to assess the amount and type of academic integrity training participants had received.

Firstly, participants were asked if they had taken courses dedicated to rules and/or ethically correct behaviour in relation to the themes introduced in the questionnaire during their current or previous studies. The answer options were: “Yes, one or more dedicated courses”, “Yes, one or more lectures”, “Yes, one or more dedicated e-learning sessions”, and “No”. Multiple answers were possible. After the data collection was complete, we identified an ambiguity in the French translation, making it difficult to distinguish between the first two options in this version of the questionnaire. As a consequence, we merged the two options in all analyses.

Secondly, participants were asked if they had learned about rules and/or ethically correct behaviour in relation to the themes introduced in the questionnaire through other means closer to daily practice. Answer options here were: “Yes, through supervisors/teachers in other courses that commented on my written work or assignments”, “Yes, through courses not dedicated exclusively to such issues”, “Yes, through discussions with fellow students”, “Yes, through discussions with senior staff outside regular courses” (for upper secondary students: “Yes, through discussions with teachers outside regular classes”), “Yes, through self-study”, “Yes, by following the procedures that are common in my field of study” (option not presented to upper secondary students), “Yes, through discussions with friends and family outside my institution”, “Yes, other”, “No”, and “I don’t know”.

4 **Questionable academic practice.** In this study, we understand questionable academic practices (QAPs) as practices that are non-compliant but not classified as academic dishonesty under the FFP definition (for a discussion of the ambiguity related to the term, see [30]). To assess the participants’ own behaviour, we presented them with a number of QAPs and asked if they had engaged in any of them during their current course of education. For each practice, the answer options were: “Yes, many times”, “Yes, a few times”, “Yes, once”, “No”, “I prefer not to answer”, “Not applicable”, and “I don’t know”. The wording and type of QAPs presented to each participant depended on their educational level and use of data. Table 6 shows the QAPs covered in this study. Two of the practices were directly inspired by [43]. For further details, see S1 File.

### 2.4. Data analysis

Descriptive statistics are presented for the measures of dedicated and practical training. For the measures focusing on direct tests of competence, rule conception, and self-reported questionable behaviour, descriptive statistics are presented across the three study levels.

Multivariable logistic regression was carried out to identify factors that are associated with the 11 measures of rule conception. These measures were treated as dependent variables and were collapsed into binary variables. For the 5 measures of rule conception that were treated as likely non-compliant (see Table 2) we assigned the response options “Yes, it is a serious violation” and “Yes, but it is not a serious violation” the value = 1, while all other response options (“The rules are unclear”, “It depends on the situation”, “No, it is not against the rules”, and “I don’t know”) received the value = 0. For the 6 measures of rule conception that were treated as likely grey-zone academic practice (see Table 2) we assigned the response options “The rules are unclear”, “It depends on the situation” the value = 1, while all other response options (“Yes, it is a serious violation” and “Yes, but it is not a serious violation”, “No, it is not against the rules”, and “I don’t know”) received the value = 0. Even though the rule conception measures have 6 response option, we decided that a collapsing into binary dependent variables was the best approach here, because the response options are categorical in nature, and no orderings can be discerned. Two variables were inserted as categorical predictors: country of study and gender. Three variables were inserted as continuous variables: age, dedicated training, and practical training. The variable dedicated training was constructed as a variable with three levels ranging from (0 = no dedicated training) to 2 (both types of dedicated training). The variable practical training was constructed as a variable with four levels ranging from 0 (no practical training received) to 3 (all three types of training were received). Regressions were run separately for each study level.

Multivariable ordered logistic regression was carried out to identify factors that were associated with the participants’ own (self-reported) questionable behaviour. The six measures of self-reported questionable behaviour were treated as dependent variables The response options of these variables received the following values in the regressions: “No” = 0, “Yes, once” = 1, “Yes, a few times” = 2, and “Yes, many times” = 3. Participants giving other responses (“I prefer not to answer”, “Not applicable” and “I don’t know”) were excluded from the analysis. The same variables (mentioned above regarding the multivariable analysis of rule conception) were inserted as predictors. These regressions were also run separately for each study level.

In supporting information (S4 File), model results are reported for all regressions carried out. We report the likelihood ratio chi2 test statistics for the full model, goodness of fit tests, and the Odds Ratio (OR) and p-value for all parameters. For the goodness of fit assessment, we use the Hosmer-Lemeshow goodness of fit test [44] to evaluate model fit in the logistic regressions. In the ordered logistic regressions, we use three diagnostics to assess goodness of fit: the ordinal Hosmer-Lemeshow test (Ordinal HL), the Pulkstenis–Robinson chi-squared tests (PR), and the Lipsitz likelihood-ratio test (Lipsitz) [45]. For all goodness of fit tests, if the p-value >0.05, we considered the regression model to possess a poor fit. This was the case in 8 of the models. In those cases, to achieve an acceptable fit, we either removed the control variables gender, age (or both), or recoded the questionable behaviour outcome variable with a poor model fit from an ordered to a binary (0 = ”no”; 1 = ”yes”). These procedures are described in the relevant regression models in supporting information S4 File.

We were particularly interested in identifying possible links between practical and dedicated training, respectively, and the students’ rule conceptions and questionable behaviours. Therefore, for all regressions where practical and dedicated training predicted conceptions and behaviours at a statistically significant level (p < 0.05) we calculated marginal effects to evaluate the direction of the association. Stata’s *margins* (dy/dx) command was used to calculate marginal effects, and the other variables in the regressions were set at their mean value (Stata’s *atmeans* sub-command). The marginal effects are presented in supporting information S4 File. In addition, we also report the magnitude of the effect from these two variables on the dependent variables where we follow Sullivan and Feinn [46] and treat OR of 1.5 as a small effect, OR of 2.0 as a medium effect, and OR of 3 as a large effect.

## 3. Results

### 3.1. Training

The data on academic integrity training show a clear gradient indicating that participants at more advanced study levels were more likely to report that they had received dedicated training somewhere along their educational trajectory (Table 3). While only 39% of upper secondary level participants said they had attended a course or lecture on academic integrity, this number was 60% for Bachelor level participants and 68% for PhD level participants. Despite this gradient following the educational trajectory, it is worth noting that many students had not received any training; the majority of upper secondary and almost one third of PhD students who participated in the study reported that they had not received any form of dedicated academic integrity training during their current or previous studies. It is also worth noting that the numbers are based on the participants’ own perception, and they may have actually received a different amount of training than reported.

**Table 3:** Dedicated academic integrity training (as perceived by participants): “Have you taken courses on rules and/or ethically correct behaviour in relation to the themes introduced above during your current or previous studies?” Share of participants who answered ‘yes’ % (n).

	Upper secondary (n = 1,260)	Bachelor (n = 922)	PhD (n = 1,115)
One or more dedicated courses or lectures	35% (437)	55% (507)	63% (699)
One or more dedicated e-sessions	6% (70)	10% (91)	10% (111)
At least one type of dedicated training	39% (495)	60% (556)	68% (761)

The data on practical training show a similar gradient indicating that participants at more advanced study levels were more likely to have received at least one type of practical training compared to participants at less advanced levels (Table 4). Among the upper secondary level participants, 59% had received practical training, whereas 72% of participants at Bachelor level and 80% of participants at PhD level reported that they had received such training. Furthermore, when comparing to Table 3, it is worth noting that more participants at all study levels reported that they had received practical compared to dedicated training.

**Table 4:** Practical academic integrity training (as perceived by participants): “Have you learned about rules and/or ethically correct behaviour in relation to the themes introduced above through any other method?” Share of participants who answered ‘yes’ %(n).

	Upper secondary (n = 1,260)	Bachelor (n = 922)	PhD (n = 1,115)
Feedback on written work or assignments in another course	41% (521)	54% (501)	62% (686)
In courses not dedicated exclusively to academic integrity	18% (232)	36% (329)	36% (399)
Through discussions with teachers/senor staff outside regular courses	17% (212)	18% (169)	40% (447)
At least one type practical training	59% (738)	72% (662)	80% (895)

### 3.2. Conceptions and competences

#### 3.2.1. Direct test of competences.

As a direct test of their competences in paraphrasing and citation practice, participants were shown four different paraphrases of a short text and asked to evaluate whether each was acceptable (see Sec. 2.3 for details).

**Fig 1:**
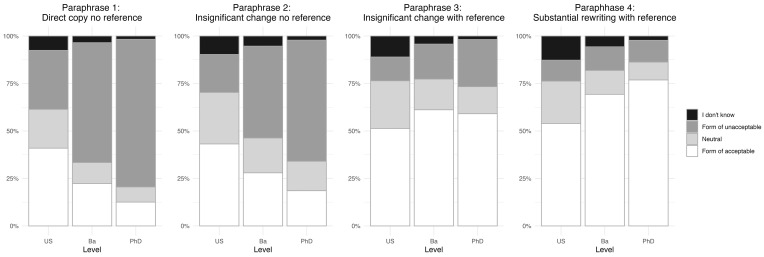
Participants’ ethical evaluation of four different paraphrases of a short text, grouped by educational level (Upper secondary (US): n = 1,260, Bachelor (Ba): n = 922, PhD: n = 1,115). The two answer options “Unacceptable” and “Completely unacceptable” are represented collectively as “Form of unacceptable”. Similarly, the answer options “Acceptable” and “Completely acceptable” are represented as “Form of acceptable”.

Once again, we see a clear gradient in the data, indicating that participants at more advanced study levels correctly classified the specific examples better than those at less advanced levels (Fig 1, see S2 File for further details). The first paraphrase (direct copy with no reference) is clearly unacceptable, yet 41% of the upper secondary level participants considered it to be either acceptable or completely acceptable, whereas only 22% and 13% of the Bachelor and PhD level participants, respectively, considered it to be so. Conversely, the fourth paraphrase is (arguably) acceptable, but only 54% of the upper secondary students were able to identify it as such, while 69% of Bachelor and 77% of PhD level participants identified it correctly.

#### 3.2.2. Rule conception.

The participants’ conceptions of the rules were tested by presenting them with 11 different academic practices (see Sec. 2.3 for details).

**Fig 2:**
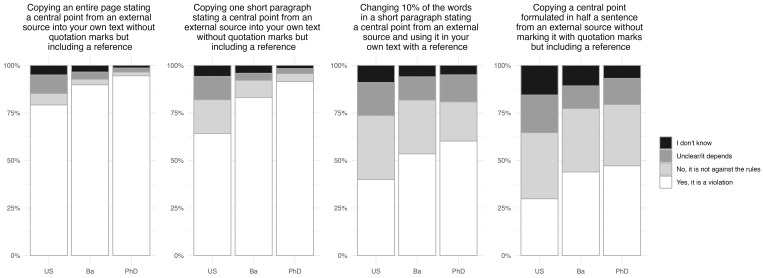
Conceptions of rules regarding citation practice, grouped by study level (Upper secondary (US): n = 1,260, Bachelor (Ba): n = 922, PhD: n = 1,115). “Please indicate whether you believe the following actions go against the official rules and regulations that apply to you in relation to plagiarism”. “Yes, it is a violation” covers the two answer options “Yes, it is a serious violation” and “Yes, but it is not a serious violation”. “Undecided/it depends” covers the two options “The rules are unclear” and “It depends on the situation”.

For the practices relating to citation and the use of others’ texts, the first two practices were likely non-compliant, whereas the last two were likely grey-zone practices (Fig 2, see S2 File for further details). Consequently, to demonstrate a good understanding of the rules, participants should choose a form of “Yes” when asked about the first two practices, and “The rules are unclear” or “It depends on the situation” when asked about the last two practices.

We see a clear trend in the data for the first two practices, such that participants at more advanced levels were more frequently able to identify them correctly as non-compliant; 79% of upper secondary level participants identified copying an entire page as non-compliant, compared to 90% of Bachelor and 95% of PhD level participants. In fact, only 54% of upper secondary level participants considered it a serious violation of the rules. Similarly, 64% of upper secondary level participants were able to identify copying a short paragraph as non-compliant, compared to 83% of Bachelor and 91% of PhD level participants.

A different picture emerges for the two grey-zone practices. Concerning the practice of changing 10% of the words in a paragraph, 17% of upper secondary level participants answered, “The rules are unclear” or “It depends on the situation”, while 12% and 14% of Bachelor and PhD level participants, respectively, chose one of these two options. Similarly, 20% of upper secondary level participants viewed copying a central point formulated in half a sentence as a grey-zone practice, compared to 12% of Bachelor and 14% of PhD level participants.

Only one of the collaborative practices included in the survey was likely non-compliant, while the other three were likely grey-zone practices (see Table 2). As PhD students would not, in our judgement, identify with these situations and understand them as part of their studies, only upper secondary and Bachelor level participants were asked these questions.

**Fig 3:**
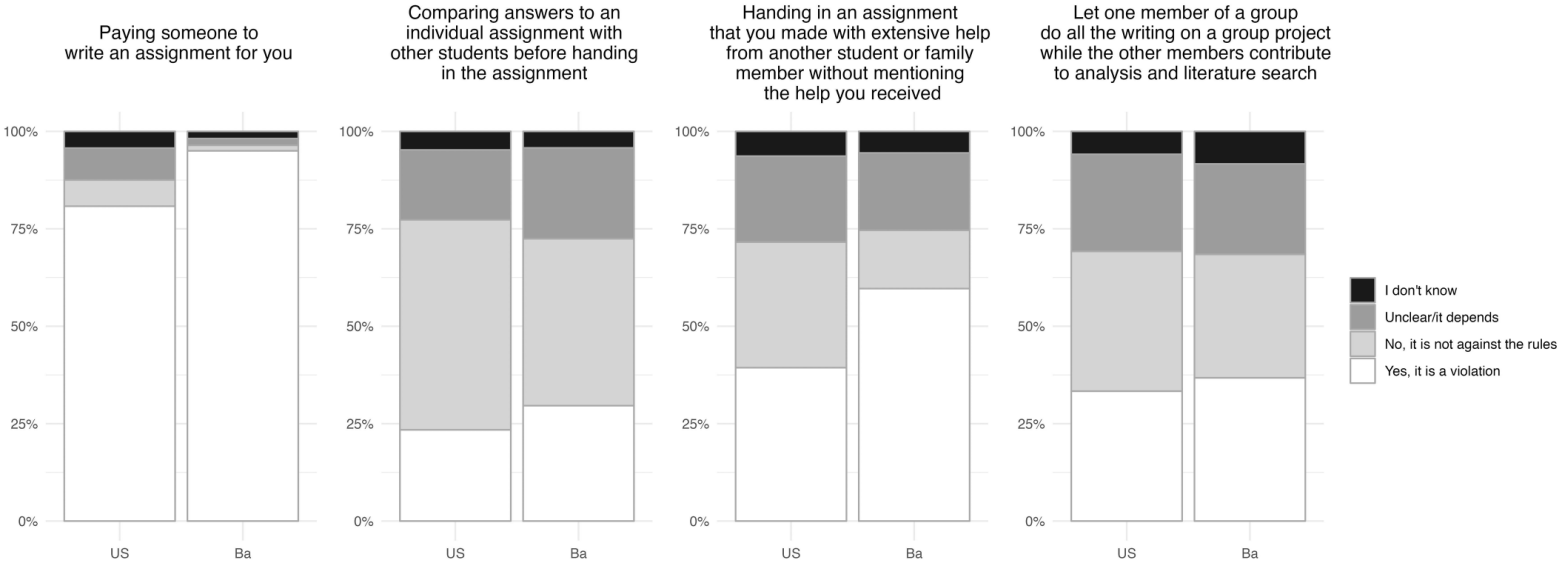
Conceptions of rules regarding collaborative practices, grouped by study level (Upper secondary (US): n = 1,260, Bachelor (Ba): n = 922). “Please indicate whether you believe the following actions go against the official rules and regulations that apply to you in relation to working with others and assigning authorship”. “Yes, it is a violation” covers the two answer options “Yes, it is a serious violation” and “Yes, but it is not a serious violation”, while “Undecided/it depends” covers the two options: “The rules are unclear” and “It depends on the situation”.

Bachelor level participants were more likely to classify the non-compliant practice of paying someone to write an assignment for you correctly (95%) by choosing one of the two ‘yes’ options than upper secondary level participants (81%; see Fig 3, see S2 File for further details). We observed little difference between the two study levels for the three grey-zone practices, with 18%, 22%, and 25% of upper secondary level participants choosing the “The rules are unclear” or “It depends on the situation”, compared to 23%, 20%, and 23% of Bachelor level participants choosing these options.

Two of the practices relating to data collection were likely non-compliant, while “Not mentioning that you removed a number of deviating data points from a dataset when the cause of the deviation was known” was likely a grey-zone practice.

**Fig 4:**
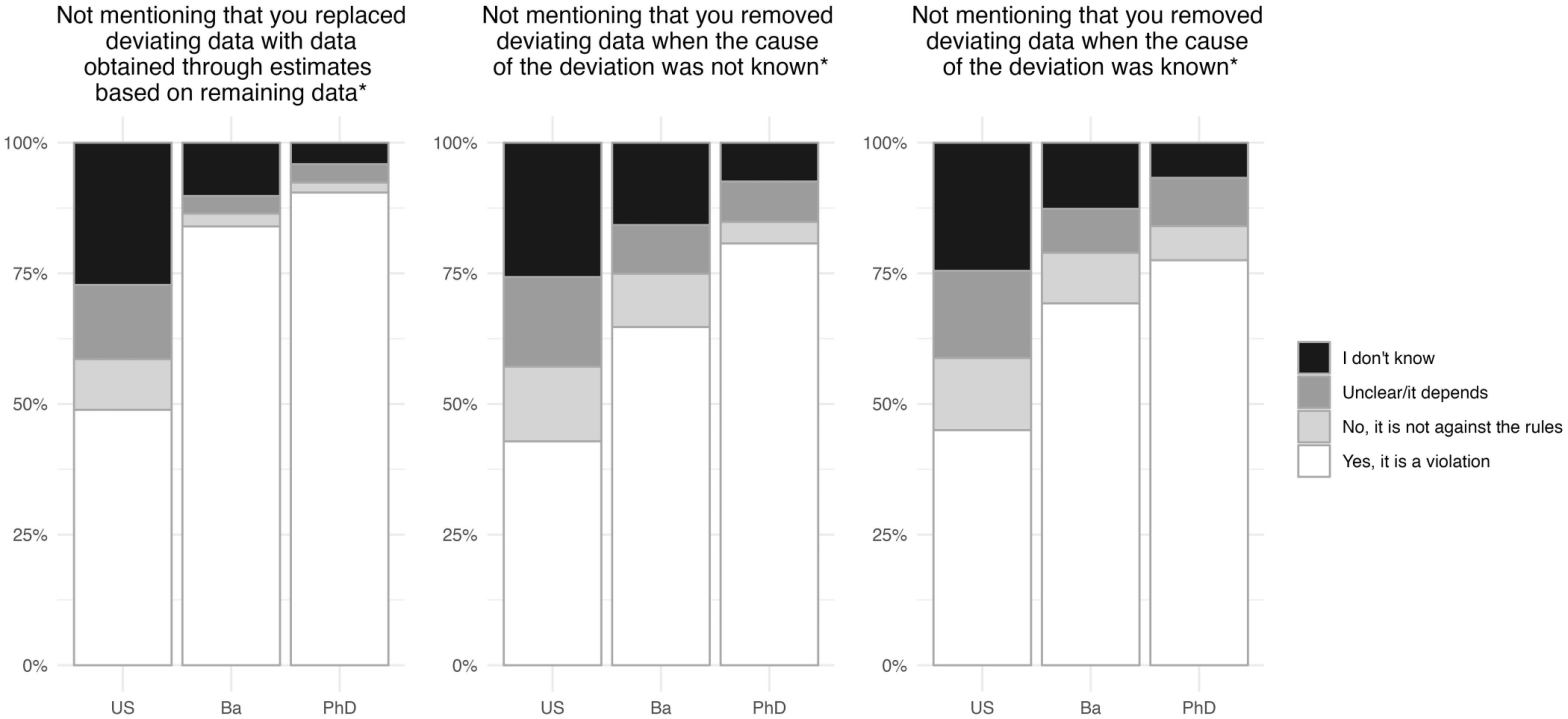
Conceptions of rules regarding data collection, grouped by study level (Upper secondary (US): n = 1,260, Bachelor (Ba): n = 774, PhD: n = 996). “Please indicate whether you believe the following actions go against the official rules and regulations that apply to you in relation to plagiarism”. Only Bachelor and PhD level participants who had indicated that they worked with data were asked this question. “Yes, it is a violation” covers the two answer options “Yes, it is a serious violation” and “Yes, but it is not a serious violation”. “Undecided/it depends” covers the two options: “The rules are unclear” and “It depends on the situation”. *Not the exact formulation of the question. See Table 5.

It is worth noting that the distribution of answers given by the upper secondary level participants was roughly the same for all three practices (Fig 4, see S2 File for further details). Furthermore, for each practice around a quarter (25% to 27%) of the participants from this group answered, “I don’t know”. This indicates that the upper secondary level participants had little or no comprehension of the integrity issues related to data handling practices. The PhD level participants were more competent in identifying the two non-compliant practices (with 90% and 81% answering a version of “yes”, respectively) than Bachelor level participants (84% and 65%). However, the two populations had roughly the same level of competence for the grey-zone practice, with 9% of PhD and 8% of Bachelor level participants answering, “The rules are unclear” or “It depends on the situation”. Therefore, the participants were once again more competent in identifying likely non-compliant than likely grey-zone practices, and there was no (noticeable) increase in grey-zone competence along the educational trajectory.

**Table 5:** Regression analysis of the conceptions of rules. The level of correct understanding for each action was tested against the five dependent variables: country, age, gender, receiving dedicated training, and receiving practical training. All correlations significant at a 5% level are listed. Cells with no significant correlations are marked with “N.S.”. Odds Ratio (OR) are reported for the two training variables where they are significant. OR for additional variables can be seen in S3 File. (US: n = 1,260; Ba: n = 922; PhD: n = 1,115).

Likely non-compliant actions						
	Copying an entire page stating a central point from an external source into your own text without quotation marks but including a reference.	Copying one short paragraph stating a central point from an external source into your own text without quotation marks but including a reference.	Paying someone to write an assignment for you.	Not mentioning in an assignment that you replaced a number of outliers in a data set with data points obtained through estimates based on the remaining data points.	Not mentioning in an assignment that you removed a number of deviating data points from a dataset when the cause of the deviation was unknown.	
US	• Practical training (p < 0.001, OR= 1.761) • Country (p = 0.022)	• Practical training (p < 0.001, OR=1.364) • Country (p < 0.001) • Gender (p = 0.014)	• Practical training (p < 0.001, OR=1.994) • Dedicated training (p = 0.037, OR=0.725) • Country (p < 0.001) • Gender (p < 0.001)	• Practical training (p < 0.001, OR=1.357)	• Practical training (p < 0.001, OR=1.442)	
Ba	• Country (p = 0.013)	• Country (p = 0.001)	• Country (p = 0.005)	• Practical training (p = 0.003, OR=1.454) • Country (p = 0.006)	Country (p = 0.003)	
Phd	• Gender (p = 0.001)	• Gender (p < 0.001)		• Dedicated training (p = 0.023, OR=1.681) • Country (p < 0.001) • Age (p = 0.048)	• Dedicated training (p = 0.001, OR=1.725) • Country (p < 0.001)	
**Likely grey-zone actions**						
	**Changing 10% of the words in a short paragraph stating a central point from an external source and using it in your own text with a reference.**	**Copying a central point formulated in half a sentence from an external source without marking it with quotation marks but including a reference.**	**Comparing answers to an individual assignment with other students before handing in the assignment.**	**Handing in an assignment that you made with extensive help from another student or family member without mentioning the help you received.**	**Let one member of a group do all the writing on a group project while the other members contribute to analysis and literature search.**	**Not mentioning in an assignment that you removed a number of deviating data points from a dataset when the cause of the deviation was known.**
US	• Gender (p = 0.006)	MODEL NOT SIGNIFICANT	MODEL NOT SIGNIFICANT	MODEL NOT SIGNIFICANT	• Gender (p = 0.026)	MODEL NOT SIGNIFICANT
Ba	MODEL NOT SIGNIFICANT	MODEL NOT SIGNIFICANT	• Country (p = 0.034)	MODEL NOT SIGNIFICANT	MODEL NOT SIGNIFICANT	MODEL NOT SIGNIFICANT
Phd	MODEL NOT SIGNIFICANT	• Age (p = 0.005)				MODEL NOT SIGNIFICANT

Regarding the association between the participants’ academic integrity training and their conceptions of rules, five general trends are worth highlighting in the results of the regression analysis (Table 5). 1) For upper secondary level participants receiving practical training was consistently correlated with a correct understanding of rules concerning likely non-compliant actions at the small to medium effect size level (OR range: 1.36–1.95). In the marginal effects analyses (S4 File 1.1−3 and 1.5-6) more practical training is consistently associated with better performance. 2) No form of training was consistently associated with Bachelor level participants’ competence. 3) There is a weak trend linking dedicated training to PhD level participants’ handling of likely non-compliant actions regarding data at the small effect size level (OR range: 1.68–1.73). More training is associated with better performance (see marginal effects analyses: S4 File 3.1–3.2). 4) As a dominant trend, the country of study was significantly linked with participants’ understanding of rules related to likely non-compliant actions in particular at the Bachelor level. 5) None of the dependent variables included in the analysis were (consistently) correlated with an understanding of rules related to likely grey-zone practices.

### 3.3. Own practice

For the participants’ own engagement in QAPs, we see a clear trend in the data, such that participants at more advanced study levels were less likely to report that they had engaged in the practices during their current studies (see Table 6). The trend can be observed for all six QAPs included in the questionnaire.

**Table 6:** Self-reported questionable behaviour: Shares of participants who answered “Yes, many times”, “Yes, a few times”, or “Yes, once”* to the question: • Upper secondary (US) students: “During your high school education, have you...” • Bachelor (Ba) students:”During your university education, have you…”• PhD students: “During your PhD, have you...” The shaded areas indicate that the question was not presented to these populations.

	US (n = 1047–1133**)	Ba (n = 665–870**)	PhD (n = 859–1046**)
Deleted or ignored deviating or unusual data based on a gut feeling that they were inaccurate or unreliable***.	48%	29%	14%
Performed a misleading or dubious interpretation or statistical analysis of data, texts, works of art, or interviews to achieve results that the teacher would accept/ a publishable result***.		18%	4%
Added students as co-authors of group assignments, even though they did not contribute.	61%	45%	
Received help from other students or family members on assignments you were supposed to complete on your own.	80%	56%	
Copied shorter passages from other sources into your own text/ research publication without marking them as quotes***.	58%	23%	8%
Kept inadequate records of parts of your work that should be documented***.		26%	20%

* For each behaviour, the sample base in the table consists of participants indicating a frequency, i.e., “Yes, many times”, “Yes, a few times” or “Yes, once”, or “No”. All other response options (“I prefer not to answer”, “Not applicable” and “I Don’t know”) were excluded.

** n is varying due to the exclusion of responses from participants answering “I prefer not to answer”, “Not applicable” and “I Don’t know”. Further, Bachelor and PhD participants who indicated they were not working with data were not presented with questions on data. See S1 File for details.

*** Not the exact wording. The wording of the questions was targeted to different groups depending on data type and study level. See S1 File for details.

It is worth noting that plagiarism (“copied shorter passages without marking them as quotes”) was a widespread practice among upper secondary level participants (58%), whereas it was significantly reduced at Bachelor level (23%) and almost eradicated at PhD level (8%). On the other hand, the two questionable collaborative practices (adding students as undeserved co-authors and receiving unauthorised assistance) were relatively common among both upper secondary (61% and 80%) and Bachelor (45% and 56%) level participants. Similarly, although the two questionable data collection practices (deleting anomalous data and keeping inadequate records) were relatively uncommon among the upper secondary (48%) and Bachelor level participants (29% and 26%), the decline in frequency along the educational trajectory is small, and these two practices were the most common questionable forms of behaviours among PhD level participants (14% and 20%).

In the analysis of possible connections between academic integrity training and engagement in QAPs (Table 7), two patterns are worth highlighting. Firstly, training is not consistently associated with engagement in the QAPs included in the survey, and where it is, the strength of the association is modest. Starting with the upper secondary level, training has a mixed link with engagement in QAP for 2 of the 4 QAPs probed about. Specifically, practical training decreases the likelihood that upper secondary participants delete or ignore unusual or deviating data, while dedicated training unexpectedly increases the likelihood that they receive help from others. Both associations are at the small effect size level (ORs: 0.73 and 1.24). For the other two study levels, the few cases where training is associated with QAP, it consistently decreases the likelihood of engagement in QAPs at the small effect size level (OR range: 0.65 and 0.80) (see further details in the marginal effects analysis S4 File). Secondly, for upper secondary and Bachelor level participants, country of study is significantly correlated with the likelihood of participants engaging in QAPs.

**Table 7:** Regression analysis of self-reported questionable behaviour. Self-reported engagement in each of the six QAPs was tested against the five background variables: country, age, gender, receiving dedicated training, and receiving practical training. All correlations significant at a 5% level are listed. Odds Ratio (OR) are reported for the two training variables where they are significant. OR for additional variables can be seen in S3 File.

	Deleted or ignored deviating or unusual data based on a gut feeling that they were inaccurate or unreliable.	Performed a misleading or dubious interpretation or statistical analysis of data, texts, works of art, or interviews to achieve results that the teacher would accept/ a publishable result.	Added students as co-authors of group assignments, even though they did not contribute.	Received help from other students or family members on assignments you were supposed to complete on your own.	Copied shorter passages from other sources into your own text/ research publication without marking them as quotes.	Kept inadequate records of parts of your work that should be documented.
US	• Practical training (p = 0.005, OR=1.274) • Country (p = 0.007) • Gender (p = 0.021)		• Country (p < 0.001) • Gender (p = 0.001)	• Dedicated training (p = 0.005, OR=1.115).	• Country (p < 0.001)	
Ba	MODEL NOT SIGNIFICANT	• Country (p = 0.007)	• Country (p < 0.001)	• Country (p < 0.001)	• Country (p < 0.001) • Dedicated training (p = 0.038, OR=0.703)	MODEL NOT SIGNIFICANT
Phd	• Practical training (p = 0.041, OR=0.802) • Dedicated training (p = 0.028, OR=0.646) • Country (p = 0.020)	MODEL NOT SIGNIFICANT			MODEL NOT SIGNIFICANT	• Country (p < 0.001) • Age (p = 0.005)

## 4. Differences between countries

The regression analyses (Tables 5 and 7) show that country of study was statistically significantly associated with conception of the rules and own engagement in questionable practices, particularly for the upper secondary and Bachelor level participants. The association was not clear for PhD level participants. It is well known that there are major differences between the educational systems in the European countries included in the survey [see, e.g., 47]. From the descriptive statistics (S2 File), we can identify clear differences in the amount of dedicated training participants from the different countries had received. In Denmark, the level of training was relatively high, where 57% of upper secondary, 72% of Bachelor and 82% of PhD level participants reported to have received some form of dedicated training. In Portugal, shares of participants who reported having received dedicated training were respectively 30%, 29% and 45% for the same three groups.

The differences were much smaller for practical training. The highest level of practical training was reported in Switzerland, where 69% of upper secondary, 80% of Bachelor and 81% of PhD level participants reported having received some form of practical training. The country with lowest level of practical training was Ireland, where the reported shares for the same three groups were respectively 49%, 74% and 82%. Interestingly, there was only little difference between the countries concerning PhD level participants’ practical training (the reported shares ranged from 79% to 82%).

As a weak trend, upper secondary and Bachelor level participants from Ireland and Portugal tended to be more in doubt about how to handle likely non-compliant actions. As an example, 25% of the Irish and 20% of the Portuguese upper secondary level participants chose one of the options “The rules are unclear”, “It depends on the situation”, or “I don’t know” when asked if it was against the rules to copy an entire page from an external source without a reference. Only 8% and 10% respectively of the Danish and Swiss upper secondary level participants chose one of these three options for this question. For Bachelor level participants shares were 7% and 12% respectively for Ireland and Portugal and 4% for both Denmark and Switzerland. As the training level is generally higher in Denmark and Switzerland for these two study levels this trend corresponds well with the results concerning likely non-compliant actions reported above (Table 5). There are, however, also examples where the trend does not hold; Swiss upper secondary level participants are for instance, more in doubt than Irish upper secondary participants about how to handle “Paying someone to write an assignment for you”.

Turning to self-reported questionable practices, there are often clear differences between the answers from the four countries involved, but there are no strong trends. As a weak trend, upper secondary and Bachelor participants from Ireland and Portugal tended to use the “I don’t know” option more than participants from the two other countries, and Bachelor level participants from Portugal generally reported to have engaged more often in the surveyed questionable practices (for four of the six practices participants from Portugal had the highest share of engagement relative to participants from the other three countries).

## 5. Discussion and conclusion

In this study we set out to provide a systematic and extensive overview of European students’ conceptions of and engagement with academic integrity and to analyse the relationship between students’ integrity-related behaviour and academic integrity training as well as demographic background variables (gender, age, country of study). Regarding the participants’ conceptions of rules, we saw a clear trend indicating that participants at more advanced study levels were more competent in identifying non-compliant practices than participants at less advanced levels (see Figs 2–4). The study was not a longitudinal study, but we designed it such that three independent groups of students were surveyed, one for each study level. As a result, we cannot determine whether individual students became more competent along their educational trajectory or if the average increase in competence at more advanced levels was due to a selection process; it may be that only the most competent students from each level progressed to the next.

Analysis of the association between academic integrity training and participants’ competence in handling non-compliant actions suggests that practical training was significantly associated with better performance for the population of upper secondary students and that dedicated training was associated with better performance for PhD students for practices related to data. No form of training was consistently associated with Bachelor level participants’ competence. When evaluating the relationship between performance and training, it is important to note that country of study was generally correlated to the participants’ competence regarding likely non-compliant practices. This result can be interpreted in several ways. For example, it may confirm the effect of contextual factors as recognised in the literature. On the other hand, we cannot rule out that the result reflect a ‘hidden’ effect of training: students in some countries may do better because they have learned about the rules during their education, even though they do not recall having done so.

The situation is markedly different for grey-zone practices, where there is no observable trend that advanced students are more competent. On the contrary, participants at all levels struggled to identify grey-zone practices, and their competences did not increase along their educational trajectory. In fact, for the two citation practices, the opposite was true: 17% and 20% of the upper secondary level participants were able to identify the examples as grey-zone practices, whereas for both practices, only 12% of the Bachelor and 14% of the PhD level participants were able to do so (Fig 2). Furthermore, none of the background variables – including training – were consistently correlated with competence concerning grey-zone practices. In short, the participants in our study had a low level of competence regarding grey-zone practices, their competences did not improve along their educational trajectory, and the academic integrity training they had received was not associated with their competences in handling these practices. Grey-zone practices simply seem to sit in a blind spot on the academic integrity map for students in higher education. We believe this is a worrying and marked result that calls for further dedicated investigation. If correct, these results will have clear implications when designing future academic integrity training. To cover the academic integrity issues that students face, the training should not only include non-compliant practices, but it should also address grey-zone practices.

If we turn to self-reported questionable practices, the positive message in the data presented above is that efforts to reduce plagiarism seem to have paid off. Participants at more advanced study levels were not only more competent in identifying non-compliant practices relating to plagiarism than participants from less advanced study levels, but they were also more competent in handling specific scenarios related to citation practice (Fig 1). More importantly, the data also showed a marked decrease in participants’ self-reported engagement in questionable practices related to plagiarism along their educational trajectory. Although 58% of upper secondary students reported that they had plagiarised shorter passages, only 8% of the PhD level participants admitted to having done so (Table 6). In other words, participants at more advanced levels had a better knowledge of the rules, were more competent in handling specific situations, and engaged less in questionable citation behaviour than students at less advanced levels.

Regarding the other two dimensions of academic integrity covered in this study, the message is less positive. Questionable collaborative practices were widespread among upper secondary and Bachelor level participants, with many admitting to granting (undeserved) ‘authorship’ credit to other students and to receiving unauthorised help (Table 6). The PhD students were not asked these questions in this way as they would not be meaningful to this population. For this reason, PhD level participants were asked a slightly different set of questions concerning authorship, reported and analysed in [28]. The results showed that more than 28% of PhD level participants said that they had “allowed research group leaders, supervisors, or others in power to become co-authors of papers, even though they did not make a significant contribution to them” at least once during their PhD studies. Although the power dynamic is radically different for the PhD students, the results in this study indicate that upper secondary and Bachelor students have few competences in handling the challenges and dilemmas related to questionable collaborative practices and that they are accustomed to breaking the rules governing this integrity dimension. If this is indeed the situation, one might expect PhD students based on their previous education to be unprepared for the challenges of authorship attribution we see in academia (for further discussion of this aspect, see [28]). This result highlights the need for a reform of integrity training, with more attention paid to issues related to collaborative practices.

Where plagiarism and questionable collaborative practices are mainly problematic because they undermine the meritocratic nature of science, questionable practices connected to data collection and analysis can be seen as attacks on the integrity of the scientific record and thus on the trustworthiness of science as an institution. In other words, questionable data practices constitute a serious threat to science, arguably more severe than the other types of questionable practices discussed here. Seen in this light, it is discouraging that upper secondary level participants seemed to have very limited competences in identifying non-compliant data collection practices, and although we see a trend in our data suggesting that questionable data collection practices were less frequent for participants at more advanced study levels, the numbers were still relatively high. Among the PhD level participants, 14% admitted to deleting deviating data “based on a gut feeling that they were inaccurate” and 20% admitted to keeping inaccurate records (Table 6). These two items in our questionnaire were inspired by similar items used in a survey of 3,247 working scientists in which a comparable proportion (15.5% and 27.5%) of the participants admitted to engaging in the practices within the last 3 years [43]. Such a high prevalence of questionable data practice is clearly worrying and, based on responses from the PhD level participants in particular, our data do not suggest that the problem has been solved or that it is even in decline. The level of training was negatively associated with PhD level participants’ engagement in the QAP related to data collection, but not with the QAPs related to data analysis and record keeping (Table 7). This result is clearly concerning and again calls for a more comprehensive approach to integrity training.

The data presented in this study have clear implications for the design of integrity training. Although it is important to address plagiarism and other clear-cut, non-compliant practices, other aspects of academic integrity deserve more attention. In particular, we saw no improvement in participants’ understanding of grey-zone practices along their educational trajectory, and although the frequency of questionable practices related to collaborative and data collection practices declined along the educational trajectory, they were still worryingly high, even among PhD level participants. It is especially concerning that dedicated training, with a few exceptions, was not associated with either better rule understanding or less engagement in QAPs. This suggests that current methods of teaching academic integrity to the groups of students surveyed in this study may not be sufficiently effective.

These results call for a revision of academic integrity training at all educational levels. We suggest that training is approached in a more encompassing way, including grey-zone situations as well as clear non-compliant actions, and a broad range of behaviours, including questions and dilemmas related to authorship, collaboration, and data collection and analysis. How these competences should be taught is an open question that clearly calls for further investigation.

As the question of educational reform falls outside the scope of this paper, we will limit ourselves to mentioning two suggestions based on the literature. First, a comprehensive meta-analysis by Katsarov and colleagues [38] suggests that engaging and experiential learning approaches tend to be more effective. This aligns well with the results reported in this study showing that practical training generally was more effective than dedicated training. As we have defined it, practical training is closer to students’ everyday practice and will thus tend to be more engaging and experiential than mere theoretical training. It is, however, an open question how such engaging practices can be implemented in dedicated training. Second, to make students better suited to handle grey-zone situations, we suggest that teachers supplement training of the basic rules with training that addresses the underlying values of scientific integrity. This could for instance, be done using a practice-near “explicit-reflective framework” as suggested by Johansen & Christiansen [30] or by applying a phronesis framework as discussed in [48]. These reforms are well aligned with theories of moral development, such as Kohlberg’s stage theory [e.g., 49], which associates moral development with a shift from rule- and authority-based reasoning toward a more principled understanding of underlying moral values. Further analysis of academic integrity based on these theories and later developments, such as models of moral behaviour [e.g., 50], may be of value when designing educational reform.

Finally, it should be noted that this study has its limitations. Our study population included groups of students from four European countries, and we cannot know if or to what extent the results are applicable to other countries. In fact, the analysis shows that country of study was a significant factor for several of the issues we addressed. For this reason, caution should be used if the results are applied to countries not included in the study. Furthermore, the study was designed to offer a comprehensive overview, and we therefore included only a small number of behaviours and situations for each of the different types. For instance, only one grey-zone situation relating to data management was included in the survey (Table 2), and important questions connected to statistical analysis such as cherry-picking and p-hacking [51] are not directly covered in the survey. This limitation was the result of a necessary trade-off, and we recommend that dedicated and more detailed studies are carried out, not least concerning p-hacking and similar questionable practices connected to the analysis of quantitative data. In addition, the effect of training was measured using the participants’ perception of the amount of training they had received, which may differ from the actual amount. It should also be noted that the regression analysis presented in Tables 5 and 7 is the result of an explorative examination involving a large number of possible connections. This kind of exploratory analysis can be misleading as there is a high risk of false positive findings. We addressed this limitation by considering only general trends rather than singular correlations in the analysis. Lastly, our study is based on self-reported data. As engagement in questionable academic practices is a socially unacceptable behaviour, we would expect the results in section 3.3 to be underreported by the participants due to social desirability bias. There are ways to mitigate this bias [e.g., 52]. However, we are not too concerned about this problem, since in this study we are mainly interested in the difference between educational levels and between various types of questionable behaviour. As far as all absolute results are affected by the bias to roughly the same extent the differences reported in our analysis will only be modestly affected by the social desirability bias. The reader should keep in mind that the shares reported in Table 6 are likely underreported. The questionable practices in question may be more common than reported.

## Supporting information

S1 File Questionnaire overview: Contains an overview of the questionnaire. (PDF)

S2 File Descriptive statistics: Contains the descriptive statistics relevant for this paper. (PDF)

S3 File Results from regression tables: Contains raw outputs tables from the regression analyses. (PDF)

S4 File Marginal effects from regression analysis: Contains marginal effects from the two training variables (‘dedicated training’ and ‘practical training’) where these were statistically significant (p < 0.05). (PDF)

S5 File Inclusivity in global research checklist. (PDF)
