# Supplementary material for: Academic integrity across educational levels: Exploring students’ engagement with grey-zone and non-compliant practices in four European countries
Source: PLoS One. 2026 Mar 4;21(3):e0342227. doi: 10.1371/journal.pone.0342227 (PMC12959713; doi:10.1371/journal.pone.0342227)
Supplement: S2 File — (PDF) [file pone.0342227.s002.pdf]

Supporting information S2: Descriptive statistics

Table 1: Dedicated academic integrity training (as perceived by participants) by study level and country:
"Have you taken courses on rules and/or ethically correct behaviour in relation to the themes introduced above during your current or previous studies?"
Share of participants who answered 'yes'

	Upper secondary	Bachelor	PhD
DENMARK	(n=389)	(n=218)	(n=427)
One or more dedicated courses or lectures	52%	66%	81%
One or more dedicated e-sessions	6%	6%	4%
At least one type of dedicated training	57%	72%	82%
IRELAND	(n=292)	(n=231)	(n=245)
One or more dedicated courses or lectures	17%	59%	65%
One or more dedicated e-sessions	8%	16%	28%
At least one type of dedicated training	24%	65%	81%
PORTUGAL	(n=219)	(n=274)	(n=241)
One or more dedicated courses or lectures	27%	27%	43%
One or more dedicated e-sessions	4%	1%	5%
At least one type of dedicated training	30%	29%	45%
SWITZERLAND*	(n=360)	(n=199)	(n=202)
One or more dedicated courses or lectures	35%	77%	45%
One or more dedicated e-sessions	4%	18%	7%
At least one type of dedicated training	38%	86%	50%

*German-speaking part

Table 2: Practical academic integrity training (as perceived by participants) by study level and country: "Have you learned about rules and/or ethically correct behaviour in relation to the themes introduced above through any other method?"
Share of participants who answered 'yes'

	Upper secondary	Bachelor	PhD
DENMARK	(n=389)	(n=218)	(n=427)
Feedback on written work or assignments in another course	44%	54%	56%
In courses not dedicated exclusively to academic integrity	7%	46%	36%
Through discussions with teachers/senor staff outside regular courses	20%	13%	49%
At least one type practical training	58%	73%	80%
IRELAND	(n=292)	(n=231)	(n=245)
Feedback on written work or assignments in another course	27%	61%	69%
In courses not dedicated exclusively to academic integrity	26%	36%	38%
Through discussions with teachers/senor staff outside regular courses	9%	19%	28%
At least one type practical training	49%	74%	82%
PORTUGAL	(n=219)	(n=274)	(n=241)
Feedback on written work or assignments in another course	44%	52%	63%
In courses not dedicated exclusively to academic integrity	12%	13%	20%
Through discussions with teachers/senor staff outside regular courses	16%	24%	37%
At least one type practical training	56%	64%	79%
SWITZERLAND*	(n=360)	(n=199)	(n=202)
Feedback on written work or assignments in another course	49%	51%	63%
In courses not dedicated exclusively to academic integrity	28%	56%	52%
Through discussions with teachers/senor staff outside regular courses	20%	16%	41%
At least one type practical training	69%	80%	81%

*German-speaking part

Table 3: Participants' ethical evaluation of four different paraphrases of a short text, grouped by educational level, shares (Upper secondary: n=1,260, Bachelor: n=922, PhD: n=1,115).

Paraphrase 1: Direct copy, no reference	Upper secondary	Bachelor	PhD
Completely acceptable	11%	6%	3%
Acceptable	30%	17%	9%
Neutral	20%	11%	8%
Unacceptable	22%	40%	48%
Completely unacceptable	9%	23%	30%
I don't know	8%	3%	2%
**Paraphrase 2: Insignificant change, no reference**	**Upper secondary**	**Bachelor**	**PhD**
Completely acceptable	10%	6%	4%
Acceptable	33%	22%	15%
Neutral	27%	18%	16%
Unacceptable	17%	36%	45%
Completely unacceptable	3%	13%	18%
I don't know	10%	5%	2%
**Paraphrase 3: Insignificant change, with a reference**	**Upper secondary**	**Bachelor**	**PhD**
Completely acceptable	17%	21%	21%
Acceptable	34%	40%	38%
Neutral	25%	16%	14%
Unacceptable	10%	17%	22%
Completely unacceptable	2%	2%	3%
I don't know	11%	4%	2%
**Paraphrase 4: substantial rewriting with a reference**	**Upper secondary**	**Bachelor**	**PhD**
Completely acceptable	24%	35%	42%
Acceptable	30%	34%	35%
Neutral	22%	13%	9%
Unacceptable	9%	10%	9%
Completely unacceptable	2%	2%	2%
I don't know	13%	6%	2%

Table 4: Conceptions of rules regarding citation practice, grouped by study level, shares within study levels (Upper secondary: n=1,260, Bachelor: n=922, PhD: n=1,115).

Scenario 1: Copying an entire page stating a central point from an external source into your own text without quotation mark but including a reference

	Upper secondary	Bachelor	PhD
Yes, it is a serious violation	54%	74%	87%
Yes, but it is not a serious violation	26%	16%	8%
No, it is not against the rules	6%	3%	2%
The rules are unclear	3%	2%	1%
It depends on the situation	7%	2%	1%
I don't know	5%	3%	1%

Scenario 2: Copying one short paragraph stating a central point from an external source into your own text without quotation marks but including a reference

	Upper secondary	Bachelor	PhD
Yes, it is a serious violation	18%	46%	66%
Yes, but it is not a serious violation	46%	37%	26%
No, it is not against the rules	18%	9%	4%
The rules are unclear	4%	1%	1%
It depends on the situation	8%	2%	2%
I don't know	6%	4%	2%

Scenario 3: Changing 10% of the words in a short paragraph stating a central point from an external source and using it in your own text with a reference

	Upper secondary	Bachelor	PhD
Yes, it is a serious violation	11%	20%	27%
Yes, but it is not a serious violation	29%	33%	33%
No, it is not against the rules	34%	28%	21%
The rules are unclear	7%	5%	6%
It depends on the situation	10%	7%	9%
I don't know	9%	6%	5%

Scenario 4: Copying a central point formulated in half a sentence from an external source without marking it with quotation marks but including a reference

	Upper secondary	Bachelor	PhD
Yes, it is a serious violation	9%	14%	17%
Yes, but it is not a serious violation	21%	30%	30%
No, it is not against the rules	35%	33%	32%
The rules are unclear	9%	6%	5%
It depends on the situation	11%	6%	8%
I don't know	16%	11%	7%

Tables 5a-5d shows conceptions of rules regarding citation practice for each country, grouped by study level, shares within study levels.

Table 5a: Scenario 1: Copying an entire page stating a central point from an external source into your own text without quotation mark but including a reference

	Yes, it is a serious violation	Yes, but it is not a serious violation	No, it is not against the rules	The rules are unclear	It depends on the situation	I don't know
Denmark						
US (n=389)	69%	17%	5%	2%	4%	3%
Ba (n=218)	80%	14%	2%	1%	0%	3%
PhD (n=427)	89%	7%	1%	1%	0%	1%
Ireland						
US (n=292)	34%	29%	11%	4%	11%	11%
Ba (n=231)	74%	16%	3%	1%	3%	3%
PhD (n=245)	88%	7%	2%	0%	2%	1%
Portugal						
US (n=219)	48%	29%	3%	4%	11%	5%
Ba (n=274)	66%	17%	4%	3%	4%	5%
PhD (n=241)	85%	7%	2%	0%	2%	3%
Switzerland*						
US (n=360)	55%	30%	4%	3%	5%	3%
Ba (n=199)	76%	17%	3%	2%	1%	2%
PhD (n=202)	83%	10%	2%	1%	2%	1%

*German-speaking part

Table 5b: Scenario 2: Copying one short paragraph stating a central point from an external source into your own text without quotation marks but including a reference

	Yes, it is a serious violation	Yes, but it is not a serious violation	No, it is not against the rules	The rules are unclear	It depends on the situation	I don't know
Denmark						
US (n=389)	22%	47%	18%	3%	6%	4%
Ba (n=218)	59%	31%	6%	0%	1%	2%
PhD (n=427)	68%	26%	3%	1%	2%	1%
Ireland						
US (n=292)	8%	40%	25%	5%	12%	10%
Ba (n=231)	46%	36%	9%	1%	4%	4%
PhD (n=245)	66%	26%	4%	0%	2%	1%
Portugal						
US (n=219)	11%	47%	18%	6%	12%	5%
Ba (n=274)	25%	50%	15%	1%	3%	7%
PhD (n=241)	63%	25%	6%	1%	1%	3%
Switzerland*						
US (n=360)	27%	48%	11%	2%	7%	4%
Ba (n=199)	60%	29%	4%	2%	2%	3%
PhD (n=202)	64%	27%	3%	1%	2%	2%

*German-speaking part

Table 5c: Scenario 3: Changing 10% of the words in a short paragraph stating a central point from an external source and using it in your own text with a reference

	Yes, it is a serious violation	Yes, but it is not a serious violation	No, it is not against the rules	The rules are unclear	It depends on the situation	I don't know
Denmark						
US (n=389)	13%	26%	33%	10%	8%	10%
Ba (n=218)	23%	30%	27%	6%	8%	5%
PhD (n=427)	22%	36%	25%	4%	9%	3%
Ireland						
US (n=292)	9%	24%	36%	5%	14%	13%
Ba (n=231)	16%	36%	28%	6%	7%	6%
PhD (n=245)	30%	34%	22%	5%	5%	3%
Portugal						
US (n=219)	7%	28%	37%	6%	13%	8%
Ba (n=274)	19%	31%	30%	3%	9%	8%
PhD (n=241)	33%	27%	15%	6%	10%	10%
Switzerland*						
US (n=360)	13%	37%	31%	7%	7%	5%
Ba (n=199)	26%	34%	27%	7%	3%	4%
PhD (n=202)	28%	33%	16%	8%	9%	5%

*German-speaking part

Table 5d: Scenario 4: Copying a central point formulated in half a sentence from an external source without marking it with quotation marks but including a reference

	Yes, it is a serious violation	Yes, but it is not a serious violation	No, it is not against the rules	The rules are unclear	It depends on the situation	I don't know
Denmark						
US (n=389)	11%	21%	31%	10%	11%	17%
Ba (n=218)	11%	30%	36%	7%	6%	9%
PhD (n=427)	13%	30%	35%	5%	11%	5%
Ireland						
US (n=292)	9%	16%	33%	9%	12%	22%
Ba (n=231)	12%	32%	32%	9%	3%	12%
PhD (n=245)	16%	33%	35%	5%	4%	7%
Portugal						
US (n=219)	7%	22%	34%	8%	15%	14%
Ba (n=274)	12%	29%	35%	4%	7%	14%
PhD (n=241)	27%	25%	26%	5%	7%	10%
Switzerland*						
US (n=360)	9%	24%	40%	8%	9%	9%
Ba (n=199)	22%	30%	30%	6%	6%	7%
PhD (n=202)	16%	30%	30%	8%	9%	7%

*German-speaking part

Table 6: Conceptions of rules regarding collaborative practices, grouped by study level, shares within study levels (Upper secondary: n=1,260, Bachelor: n=922).

Scenario 1: Paying someone to write an assignment for you.

	Upper secondary	Bachelor	PhD
Yes, it is a serious violation	72%	94%	
Yes, but it is not a serious violation	9%	1%	
No, it is not against the rules	7%	1%	
The rules are unclear	4%	1%	
It depends on the situation	4%	1%	
I don't know	4%	2%	

Scenario 2: Comparing answers to an individual assignment with other students before handing in the assignment.

	Upper secondary	Bachelor	PhD
Yes, it is a serious violation	7%	12%	
Yes, but it is not a serious violation	16%	17%	
No, it is not against the rules	54%	43%	
The rules are unclear	7%	9%	
It depends on the situation	11%	15%	
I don't know	5%	4%	

Scenario 3: Handing in an assignment that you made with extensive help from another student or family member, without mentioning the help you received.

	Upper secondary	Bachelor	PhD
Yes, it is a serious violation	13%	35%	
Yes, but it is not a serious violation	26%	25%	
No, it is not against the rules	32%	15%	
The rules are unclear	10%	9%	
It depends on the situation	12%	11%	
I don't know	6%	6%	

Scenario 4: Letting one member of a group do all the writing on a group project while the other members contribute to the analysis and literature search.

	Upper secondary	Bachelor	PhD
Yes, it is a serious violation	14%	17%	
Yes, but it is not a serious violation	20%	20%	
No, it is not against the rules	36%	32%	
The rules are unclear	8%	10%	
It depends on the situation	17%	14%	
I don't know	6%	8%	

Tables 7a-7d shows conceptions of rules regarding collaborative practice for each country, grouped by study level, shares within study levels.

Table 7a: Scenario 1: Paying someone to write an assignment for you

	Yes, it is a serious violation	Yes, but it is not a serious violation	No, it is not against the rules	The rules are unclear	It depends on the situation	I don't know
Denmark						
US (n=389)	84%	6%	3%	2%	2%	4%
Ba (n=218)	98%	0%	0%	0%	0%	1%
Ireland						
US (n=292)	74%	8%	6%	3%	4%	5%
Ba (n=231)	96%	0%	0%	0%	1%	2%
Portugal						
US (n=219)	62%	10%	11%	3%	9%	5%
Ba (n=274)	88%	2%	3%	2%	2%	2%
Switzerland*						
US (n=360)	64%	13%	9%	8%	4%	4%
Ba (n=199)	93%	3%	1%	1%	0%	2%

*German-speaking part

Table 7b: Scenario 2: Comparing answers to an individual assignment with other students before handing in the assignment

	Yes, it is a serious violation	Yes, but it is not a serious violation	No, it is not against the rules	The rules are unclear	It depends on the situation	I don't know
Denmark						
US (n=389)	4%	12%	60%	7%	11%	5%
Ba (n=218)	12%	14%	40%	9%	22%	4%
Ireland						
US (n=292)	17%	23%	34%	6%	13%	6%
Ba (n=231)	21%	19%	32%	11%	13%	5%
Portugal						
US (n=219)	8%	16%	51%	7%	13%	5%
Ba (n=274)	8%	18%	51%	8%	11%	4%
Switzerland*						
US (n=360)	2%	14%	65%	7%	8%	4%
Ba (n=199)	9%	17%	48%	7%	14%	5%

*German-speaking part

Table 7c: Scenario 3: Handing in an assignment that you made with extensive help from another student or family member, without mentioning the help you received

	Yes, it is a serious violation	Yes, but it is not a serious violation	No, it is not against the rules	The rules are unclear	It depends on the situation	I don't know
Denmark						
US (n=389)	8%	22%	39%	10%	12%	8%
Ba (n=218)	31%	22%	17%	8%	13%	9%
Ireland						
US (n=292)	18%	32%	21%	9%	12%	8%
Ba (n=231)	47%	23%	11%	11%	4%	3%
Portugal						
US (n=219)	19%	28%	26%	10%	14%	4%
Ba (n=274)	31%	27%	15%	9%	12%	5%
Switzerland*						
US (n=360)	11%	24%	38%	12%	10%	5%
Ba (n=199)	31%	26%	17%	9%	13%	5%

*German-speaking part

Table 7d: Scenario 4: Letting one member of a group do all the writing on a group project while the other members contribute to the analysis and literature search

	Yes, it is a serious violation	Yes, but it is not a serious violation	No, it is not against the rules	The rules are unclear	It depends on the situation	I don't know
Denmark						
US (n=389)	17%	24%	26%	11%	15%	6%
Ba (n=218)	17%	26%	25%	9%	15%	9%
Ireland						
US (n=292)	16%	24%	34%	5%	14%	7%
Ba (n=231)	17%	21%	33%	12%	9%	9%
Portugal						
US (n=219)	20%	19%	27%	8%	21%	5%
Ba (n=274)	24%	22%	26%	6%	17%	6%
Switzerland*						
US (n=360)	5%	11%	53%	9%	17%	4%
Ba (n=199)	7%	11%	46%	13%	14%	10%

*German-speaking part

Table 8: Conceptions of rules regarding data collection, grouped by study level, shares within study levels (Upper secondary: n=1,260, Bachelor: n=774, PhD: n=996).

Scenario 1: Not mentioning in an assignment/paper that you replaced a number of outliers in a dataset with data points obtained through estimates based on the remaining data points.

	Upper secondary	Bachelor	PhD
Yes, it is a serious violation	26%	69%	81%
Yes, but it is not a serious violation	23%	15%	9%
No, it is not against the rules	10%	2%	2%
The rules are unclear	8%	2%	1%
It depends on the situation	7%	2%	3%
I don't know	27%	10%	4%

Scenario 2: Not mentioning in an assignment/paper that you removed a number of deviating data points from a dataset when the cause of the deviation was unknown.

	Upper secondary	Bachelor	PhD
Yes, it is a serious violation	20%	43%	67%
Yes, but it is not a serious violation	23%	22%	14%
No, it is not against the rules	14%	10%	4%
The rules are unclear	10%	5%	3%
It depends on the situation	8%	5%	5%
I don't know	26%	16%	7%

Scenario 3: Not mentioning in an assignment/paper that you removed a number of deviating data points from a dataset when the cause of the deviation was known.

	Upper secondary	Bachelor	PhD
Yes, it is a serious violation	21%	45%	61%
Yes, but it is not a serious violation	24%	24%	17%
No, it is not against the rules	14%	10%	7%
The rules are unclear	9%	4%	3%
It depends on the situation	8%	5%	6%
I don't know	25%	13%	7%

Tables 9a-c shows conceptions of rules regarding data collection for each country, grouped by study level, shares within study levels.

Table 9a: Scenario 1: Not mentioning in an assignment/paper that you replaced a number of outliers in a dataset with data points obtained through estimates based on the remaining data points.

	Yes, it is a serious violation	Yes, but it is not a serious violation	No, it is not against the rules	The rules are unclear	It depends on the situation	I don't know
Denmark						
US (n=389)	26%	25%	9%	8%	5%	26%
BA (n=184)	76%	13%	3%	1%	2%	5%
PhD (n=392)	88%	9%	1%	1%	1%	1%
Ireland						
US (n=292)	21%	23%	11%	5%	5%	36%
BA (n=201)	62%	19%	2%	2%	1%	12%
PhD (n=220)	84%	9%	2%	0%	1%	4%
Portugal						
US (n=219)	23%	24%	12%	8%	13%	21%
BA (n=229)	57%	19%	3%	2%	2%	17%
PhD (n=218)	64%	12%	5%	2%	7%	10%
Switzerland*						
US (n=360)	33%	20%	8%	9%	5%	25%
BA (n=160)	85%	8%	1%	2%	1%	3%
PhD (n=166)	86%	7%	0%	1%	2%	4%

*German-speaking part

Table 9b: Scenario 2: Not mentioning in an assignment/paper that you removed a number of deviating data points from a dataset when the cause of the deviation was unknown.

	Yes, it is a serious violation	Yes, but it is not a serious violation	No, it is not against the rules	The rules are unclear	It depends on the situation	I don't know
Denmark						
US (n=389)	19%	24%	15%	9%	9%	24%
BA (n=184)	47%	22%	10%	5%	7%	9%
PhD (n=392)	74%	12%	2%	1%	6%	5%
Ireland						
US (n=292)	21%	22%	10%	8%	4%	35%
BA (n=201)	36%	26%	11%	6%	3%	16%
PhD (n=220)	73%	12%	4%	3%	2%	7%
Portugal						
US (n=219)	19%	16%	21%	10%	12%	22%
BA (n=229)	36%	19%	11%	4%	4%	25%
PhD (n=218)	45%	19%	9%	4%	9%	14%
Switzerland*						
US (n=360)	22%	26%	13%	12%	6%	21%
BA (n=160)	55%	22%	7%	2%	6%	9%
PhD (n=166)	69%	13%	5%	4%	3%	6%

*German-speaking part

**Table 9c:** Scenario 3: Not mentioning in an assignment/paper that you removed a number of deviating data points from a dataset when the cause of the deviation was known.						
	Yes, it is a serious violation	Yes, but it is not a serious violation	No, it is not against the rules	The rules are unclear	It depends on the situation	I don't know
Denmark						
US (n=389)	18%	33%	12%	7%	8%	22%
BA (n=184)	47%	26%	10%	4%	4%	10%
PhD (n=392)	59%	19%	7%	1%	8%	5%
Ireland						
US (n=292)	21%	23%	9%	7%	7%	33%
BA (n=201)	43%	24%	9%	4%	4%	15%
PhD (n=220)	64%	16%	6%	5%	4%	5%
Portugal						
US (n=219)	20%	17%	21%	9%	12%	21%
BA (n=229)	45%	19%	8%	4%	7%	16%
PhD (n=218)	54%	16%	7%	3%	8%	12%
Switzerland*						
US (n=360)	24%	21%	15%	11%	7%	22%
BA (n=160)	48%	28%	13%	1%	4%	8%
PhD (n=166)	67%	14%	4%	4%	5%	5%

*German-speaking part

Table 10a-f shows self-reported questionable behaviour for each country, grouped by study level, shares within study levels.

- Upper secondary (US) students: "During your high school education, have you..."
- Bachelor (Ba) students: "During your university education, have you…"
- PhD students: "During your PhD, have you..."

Table 10a: Deleted or ignored deviating or unusual data based on a gut feeling that they were inaccurate or unreliable

	Yes, many times	Yes, a few times	Yes, once	No	Not applicable	I prefer not to answer	I don't know
Denmark							
US (n=389)	3%	14%	15%	53%	2%	2%	10%
Ba (n=184)	1%	7%	15%	66%	4%	1%	7%
PhD (n=392)	0%	3%	6%	85%	5%	0%	1%
Ireland							
US (n=292)	6%	18%	15%	35%	2%	1%	22%
Ba (n=201)	1%	9%	15%	59%	6%	2%	6%
PhD (n=220)	0%	6%	10%	75%	5%	0%	3%
Portugal							
US (n=219)	6%	23%	15%	37%	4%	3%	13%
Ba (n=229)	0%	16%	13%	55%	6%	1%	8%
PhD (n=218)	1%	5%	8%	74%	9%	0%	2%
Switzerland*							
US (n=360)	3%	19%	24%	42%	2%	1%	8%
Ba (n=160)	0%	8%	13%	65%	10%	0%	5%
PhD (n=166)	1%	4%	8%	76%	8%	1%	2%

*German-speaking part

Table 10b: Performed a misleading or dubious interpretation or statistical analysis of data, texts, works of art, or interviews to achieve results that the teacher would accept / a publishable result

	Yes, many times	Yes, a few times	Yes, once	No	Not applicable	I prefer not to answer	I don't know
Denmark							
Ba (n=184)	0%	5%	10%	78%	2%	0%	4%
PhD (n=392)	0%	0%	3%	89%	6%	0%	1%
Ireland							
Ba (n=201)	1%	6%	12%	70%	6%	1%	3%
PhD (n=220)	0%	3%	2%	89%	4%	0%	1%
Portugal							
Ba (n=229)	0%	8%	11%	68%	5%	1%	7%
PhD (n=218)	0%	0%	2%	84%	9%	1%	3%
Switzerland*							
Ba (n=160)	0%	2%	6%	83%	7%	0%	2%
PhD (n=166)	0%	1%	1%	89%	7%	1%	2%

*German-speaking part

**Table 10c:** Added students as co-authors of group assignments, even though they did not contribute.							
	Yes, many times	Yes, a few times	Yes, once	No	Not applicable	I prefer not to answer	I don't know
Denmark							
US (n=389)	19%	32%	16%	21%	1%	3%	7%
Ba (n=218)	3%	17%	22%	52%	3%	1%	2%
Ireland							
US (n=292)	12%	21%	12%	35%	2%	2%	16%
Ba (n=231)	3%	13%	15%	59%	7%	1%	0%
Portugal							
US (n=219)	14%	29%	12%	30%	2%	4%	10%
Ba (n=274)	10%	35%	17%	29%	5%	1%	3%
Switzerland*							
US (n=360)	10%	21%	13%	48%	3%	2%	3%
Ba (n=199)	4%	8%	10%	71%	7%	1%	1%

*German-speaking part

**Table 10d:** Received help from other students or family members on assignments you were supposed to complete on your own.							
	Yes, many times	Yes, a few times	Yes, once	No	Not applicable	I prefer not to answer	I don't know
Denmark							
US (n=289)	14%	42%	17%	18%	1%	3%	5%
Ba (n=218)	4%	22%	14%	53%	2%	1%	5%
Ireland							
US (n=292)	24%	35%	11%	17%	0%	2%	11%
Ba (n=231)	8%	29%	20%	39%	1%	0%	2%
Portugal							
US (n=219)	15%	45%	13%	15%	3%	3%	6%
Ba (n=274)	5%	43%	15%	31%	3%	1%	1%
Switzerland*							
US (n=360)	14%	43%	15%	21%	2%	2%	4%
Ba (n=199)	5%	28%	17%	44%	3%	0%	3%

*German-speaking part

Table 10e: Copied shorter passages from other sources into your own text / research publication without marking them as quotes

	Yes, many times	Yes, a few times	Yes, once	No	Not applicable	I prefer not to answer	I don't know
Denmark							
US (n=389)	6%	19%	19%	45%	1%	2%	8%
Ba (n=218)	0%	6%	6%	82%	2%	1%	4%
PhD (n=427)	0%	2%	4%	87%	6%	0%	1%
Ireland							
US (n=292)	18%	31%	14%	21%	0%	2%	14%
Ba (n=231)	3%	7%	13%	70%	1%	1%	5%
PhD (n=245)	0%	5%	5%	85%	2%	0%	2%
Portugal							
US (n=219)	10%	37%	14%	24%	4%	3%	8%
Ba (n=274)	2%	21%	13%	57%	2%	1%	5%
PhD (n=241)	1%	4%	4%	85%	5%	0%	1%
Switzerland*							
US (n=360)	4%	19%	17%	48%	1%	3%	8%
Ba (n=199)	2%	4%	5%	82%	5%	1%	2%
PhD (n=202)	0%	2%	2%	89%	3%	1%	1%

*German-speaking part

Table 10f: Kept inadequate records of parts of your work that should be documented.

	Yes, many times	Yes, a few times	Yes, once	No	Not applicable	I prefer not to answer	I don't know
Denmark							
Ba (n=)	1%	13%	9%	59%	8%	0%	11%
PhD (n=)	0%	9%	10%	71%	6%	1%	4%
Ireland							
Ba (n=)	4%	12%	10%	57%	7%	0%	9%
PhD (n=)	2%	15%	9%	62%	7%	0%	4%
Portugal							
Ba (n=)	1%	9%	7%	55%	10%	0%	19%
PhD (n=)	2%	1%	6%	74%	12%	0%	4%
Switzerland*							
Ba (n=)	1%	7%	11%	63%	6%	0%	13%
PhD (n=)	2%	7%	5%	66%	11%	1%	8%

*German-speaking part
