# Supplementary material for: Academic integrity across educational levels: Exploring students’ engagement with grey-zone and non-compliant practices in four European countries
Source: PLoS One. 2026 Mar 4;21(3):e0342227. doi: 10.1371/journal.pone.0342227 (PMC12959713; doi:10.1371/journal.pone.0342227)
Supplement: S3 File — (PDF) [file pone.0342227.s003.pdf]

Supporting information S3: Results from regression tables

Results from all regression analyses including omnibus model tests and goodness of fit results are reported below. Results from each educational level (upper secondary. bachelor and PhD) are reported separately.

Upper secondary level

Table 1.1
Dependent variable: ”Copying an entire page stating a central point from an external source into your own text without quotation marks but including a reference.”
Omnibus model test: Likelihood ratio chi2(8)=98.98; p<0.001; n=1260.
Goodness of fit result: Hosmer-Lemeshow Goodness-of-fit test: chi2(8) = 11.45; p =0.1773.

		Hypothesis Test		
**Parameter**	**OR**	**Wald Chi-Square**	**df**	**Sig.**
(Intercept)	0.788	0.017	1	0.898
Switzerland	0.758	1.605	1	0.205
Portugal	0.607	3.685	1	0.055
Ireland	0.344	9.036	1	0.003
Denmark (reference)	1[a]			
Other/don't want to tell	0.869	0.328	1	0.567
Female	1.316	3.030	1	0.082
Male (reference)	1[a]			
Age (in years)	1.097	0.862	1	0.353
Practical Training	1.761	26.884	1	<0.001
Dedicated Training	0.823	1.678	1	0.195

a. Set to 1 because this parameter is redundant.

**Table 1.2**<br>Dependent variable: ”Copying one short paragraph stating a central point from an external source into your own text without quotation marks but including a reference.”<br>Omnibus model test: Likelihood ratio chi2(8)= 84.78; p<0.001 ; n=1260.<br>Goodness of fit result: Hosmer-Lemeshow Goodness-of-fit test: chi2(8)=5.13; p=0.7440.				
		**Hypothesis Test**		
**Parameter**	**OR**	**Wald Chi-Square**	**df**	**Sig.**
(Intercept)	1.108	0.015	1	0.903
Switzerland	1.227	1.454	1	0.228
Portugal	0.645	5.220	1	0.022
Ireland	0.429	15.550	1	<0.001
Denmark (reference)	1[a]			
Other/don't want to tell	1.053	0.058	1	0.810
Female	1.449	7.792	1	0.005
Male (reference)	1[a]			
Age (in years)	1.018	0.163	1	0.686
Practical Training	1.364	13.540	1	<0.001
Dedicated Training	0.920	0.439	1	0.507

a. Set to 1 because this parameter is redundant.

**Table 1.3**<br>Dependent variable: ”Changing 10% of the words in a short paragraph stating a central point from an external source and using it in your own text with a reference.”<br>Omnibus model test: Likelihood ratio chi2(8)= 15.85; p=0.0446; n=1260.<br>Goodness of fit result: Hosmer-Lemeshow Goodness-of-fit test: chi2(8)=7.75; p=0.4584.				
		**Hypothesis Test**		
**Parameter**	**OR**	**Wald Chi-Square**	**df**	**Sig.**
(Intercept)	0.273	2.286	1	0.131
Switzerland	0.711	2.702	1	0.100
Portugal	0.976	0.011	1	0.918
Ireland	1.038	0.022	1	0.882
Denmark (reference)	1[a]			
Other/don't want to tell	1.528	3.068	1	0.080
Female	0.746	3.154	1	0.076
Male (reference)	1[a]			
Age (in years)	1.002	0.001	1	0.973
Practical Training	0.957	0.189	1	0.663
Dedicated Training	0.856	0.978	1	0.323

a. Set to 1 because this parameter is redundant.

**Table 1.4**<br>Dependent variable: ”Copying a central point formulated in half a sentence from an external source without marking it with quotation marks but including a reference.”<br>Omnibus model test: Likelihood ratio chi2(8)=13.40 ; p=0.0988; n=1260.<br>Goodness of fit result: Hosmer-Lemeshow Goodness-of-fit test: chi2(8)= 7.81; p=0.4518.				
		**Hypothesis Test**		
**Parameter**	**OR**	**Wald Chi-Square**	**df**	**Sig.**
(Intercept)	1.677	0.088	1	0.767
Switzerland	0.873	0.484	1	0.487
Portugal	1.017	0.005	1	0.945
Ireland	0.772	0.577	1	0.448
Denmark (reference)	1[a]			
Other/don't want to tell	0.980	0.007	1	0.934
Female	0.763	3.035	1	0.081
Male (reference)	1[a]			
Age (in years)	0.917	0.858	1	0.354
Practical Training	0.800	5.013	1	0.025
Dedicated Training	1.060	0.156	1	0.693

a. Set to 1 because this parameter is redundant.

**Table 1.5**<br>Dependent variable: ”Paying someone to write an assignment for you.”<br>Omnibus model test: Likelihood ratio chi2(8)=135.74 ; p<0.001; n=1260.<br>Goodness of fit result: Hosmer-Lemeshow Goodness-of-fit test: chi2(8)= 12.20; p=0.1425.				
		**Hypothesis Test**		
**Parameter**	**OR**	**Wald Chi-Square**	**df**	**Sig.**
(Intercept)	7.776	6.914	1	0.009
Switzerland	0.267	34.876	1	<0.001
Portugal	0.266	28.228	1	<0.001
Ireland	0.406	10.862	1	0.001
Denmark (reference)	1[a]			
Other/don't want to tell	0.670	3.103	1	0.078
Female	2.330	25.917	1	<0.001
Male (reference)	1[a]			
Age (in years)	0.977	0.333	1	0.564
Practical Training	1.994	36.459	1	<0.001
Dedicated Training	0.725	4.337	1	0.037

a. Set to 1 because this parameter is redundant.

**Table 1.6**<br>Dependent variable: ”Comparing answers to an individual assignment with other students before handing in the assignment.”<br>Omnibus model test: Likelihood ratio chi2(8)=12.96; p=0.1133; n=1260.<br>Goodness of fit result: Hosmer-Lemeshow Goodness-of-fit test: chi2(8)=5.03; p=0.7549.				
		**Hypothesis Test**		
**Parameter**	**OR**	**Wald Chi-Square**	**df**	**Sig.**
(Intercept)	1.592	0.062	1	0.803
Switzerland	0.771	1.601	1	0.206
Portugal	0.865	0.324	1	0.569
Ireland	0.743	0.685	1	0.408
Denmark (reference)	1[a]			
Other/don't want to tell	1.629	4.019	1	0.045
Female	0.902	0.391	1	0.532
Male (reference)	1[a]			
Age (in years)	0.909	0.915	1	0.339
Practical Training	0.928	0.543	1	0.461
Dedicated Training	0.825	1.512	1	0.219

a. Set to 1 because this parameter is redundant.

**Table 1.7**<br>Dependent variable: ”Handing in an assignment that you made with extensive help from another student or family member without mentioning the help you received.”<br>Omnibus model test: Likelihood ratio chi2(8)=5.51; p=0.7022; n=1260.<br>Goodness of fit result: Hosmer-Lemeshow Goodness-of-fit test: chi2(8)=8.22; p= 0.4122.				
		**Hypothesis Test**		
**Parameter**	**OR**	**Wald Chi-Square**	**df**	**Sig.**
(Intercept)	2.431	0.263	1	0.608
Switzerland	1.068	0.130	1	0.718
Portugal	0.960	0.030	1	0.863
Ireland	0.691	1.210	1	0.271
Denmark (reference)	1[a]			
Other/don't want to tell	0.964	0.023	1	0.881
Female	0.933	0.214	1	0.644
Male (reference)	1[a]			
Age (in years)	0.888	1.628	1	0.202
Practical Training	0.954	0.266	1	0.606
Dedicated Training	1.269	2.949	1	0.086

a. Set to 1 because this parameter is redundant.

**Table 1.8**<br>Dependent variable: ”Let one member of a group do all the writing on a group project while the other members contribute to analysis and literature search.”<br>Omnibus model test: Likelihood ratio chi2(8)=16.91; p=0.0311; n=1260.<br>Goodness of fit result: Hosmer-Lemeshow Goodness-of-fit test: chi2(8)=10.04; p=0.2622.				
		**Hypothesis Test**		
**Parameter**	**OR**	**Wald Chi-Square**	**df**	**Sig.**
(Intercept)	0.140	5.654	1	0.017
Switzerland	0.971	0.029	1	0.866
Portugal	1.153	0.487	1	0.485
Ireland	0.804	0.862	1	0.353
Denmark (reference)	1[a]			
Other/don't want to tell	1.557	4.033	1	0.045
Female	0.881	0.751	1	0.386
Male (reference)	1[a]			
Age (in years)	1.049	1.230	1	0.267
Practical Training	1.082	0.819	1	0.366
Dedicated Training	1.001	<0.001	1	0.994

a. Set to 1 because this parameter is redundant.

**Table 1.9**<br>Dependent variable: ”Not mentioning in an assignment that you replaced a number of outliers in a data set with data points obtained through estimates based on the remaining data points.”<br>Omnibus model test: Likelihood ratio chi2(8)=29.00; p<0.001; n=1260.<br>Goodness of fit result: Hosmer-Lemeshow Goodness-of-fit test: chi2(8)=10.29; p=0.2454.				
		**Hypothesis Test**		
**Parameter**	**OR**	**Wald Chi-Square**	**df**	**Sig.**
(Intercept)	1.433	0.218	1	0.640
Switzerland	1.043	0.076	1	0.783
Portugal	0.849	0.797	1	0.372
Ireland	0.698	3.131	1	0.077
Denmark (reference)	1[a]			
Other/don't want to tell	0.702	2.842	1	0.092
Female	0.971	0.055	1	0.814
Male (reference)	1[a]			
Age (in years)	0.972	0.501	1	0.479
Practical Training	1.357	15.536	1	<0.001
Dedicated Training	1.051	0.181	1	0.671

a. Set to 1 because this parameter is redundant.

**Table 1.10**<br>Dependent variable: “Not mentioning in an assignment that you removed a number of deviating data points from a dataset when the cause of the deviation was unknown”<br>Omnibus model test: Likelihood ratio chi2(8)=38.35; p<0.001; n=1260.<br>Goodness of fit result: Hosmer-Lemeshow Goodness-of-fit test: chi2(8)=9.62; p=0.2930.				
		**Hypothesis Test**		
**Parameter**	**OR**	**Wald Chi-Square**	**df**	**Sig.**
(Intercept)	0.698	0.277	1	0.599
Switzerland	1.142	0.763	1	0.382
Portugal	0.733	2.734	1	0.098
Ireland	0.990	0.003	1	0.958
Denmark (reference)	1[a]			
Other/don't want to tell	0.615	4.950	1	0.026
Female	0.880	1.005	1	0.316
Male (reference)	1[a]			
Age (in years)	0.996	0.012	1	0.913
Practical Training	1.442	22.046	1	<0.001
Dedicated Training	0.981	0.025	1	0.874

a. Set to 1 because this parameter is redundant.

**Table 1.11**<br>Dependent variable: ”Not mentioning in an assignment that you removed a number of deviating data points from a dataset when the cause of the deviation was known.”<br>Omnibus model test: Likelihood ratio chi2(8)=12.16; p=0.1442; n=1260.<br>Goodness of fit result: Hosmer-Lemeshow Goodness-of-fit test: chi2(8)=3.19; p=0.9216.				
		**Hypothesis Test**		
**Parameter**	**OR**	**Wald Chi-Square**	**df**	**Sig.**
(Intercept)	0,213	3.248	1	0.071
Switzerland	1,249	1.225	1	0.268
Portugal	1,362	1.753	1	0.185
Ireland	0,832	0.473	1	0.491
Denmark (reference)	1[a]			
Other/don't want to tell	1,106	0.157	1	0.692
Female	0,815	1.507	1	0.220
Male (reference)	1[a]			
Age (in years)	1,005	0.011	1	0.918
Practical Training	0,822	3.481	1	0.062
Dedicated Training	1,038	0.057	1	0.812

a. Set to zero because this parameter is redundant.

Table 1.12
Dependent variable: "Deleted or ignored deviating or unusual data based on a gut feeling that they were inaccurate or unreliable."
<u>Original model</u>
Omnibus model test: Likelihood ratio chi2(8)=37.60; p<0.001 ; n=1047.
Goodness of fit result: Ordinal HL=0.3094; PR p=0.0128; Lipsitz=0.2147.
<u>New model</u>
Outcome variable recoded to a binary variable to obtain acceptable goodness of fit.
Omnibus model test: Likelihood ratio chi2(8)=39.27; p<0.001 ; n=1047.
Goodness of fit result: Hosmer-Lemeshow Goodness-of-fit test: chi2(8)=5.24; p=0.7317.

		Hypothesis Test		
**Parameter**	**OR**	**Wald Chi-Square**	**df**	**Sig.**
(Intercept)	0.812	0.051	1	0.821
Switzerland	1.667	9.667	1	0.002
Portugal	1.663	6.072	1	0.014
Ireland	1.692	5.014	1	0.025
Denmark (reference)	1[a]			
Other/don't want to tell	1.952	7.728	1	0.005
Female	1.142	0.912	1	0.339
Male (reference)	1[a]			
Age (in years)	0.974	0.292	1	0.589
Practical Training	1.274	8.046	1	0.005
Dedicated Training	0.820	2.267	1	0.132

a. Set to 1 because this parameter is redundant.

**Table 1.13**<br>Dependent variable: "Added students as co-authors of group assignments, even though they did not contribute."<br>Omnibus model test: Likelihood ratio chi2(8)=79.35; p<0.001; n=1096.<br>Goodness of fit result: Ordinal HL=0.3459; PR p=0.5337; Lipsitz=0.3163.				
		**Hypothesis Test**		
**Parameter**	**OR**	**Wald Chi-Square**	**df**	**Sig.**
Threshold 1	0.190	3.855	1	0.050
Threshold 2	0.376	1.342	1	0.247
Threshold 3	1.772	0.458	1	0.498
Switzerland	0.336	54.687	1	<0.001
Portugal	0.624	6.884	1	0.009
Ireland	0.401	18.886	1	<0.001
Denmark (reference)	1[a]			
Other/don't want to tell	1.745	6.813	1	0.009
Female	1.575	13.207	1	<0.001
Male (reference)	1[a]			
Age (in years)	0.953	1.161	1	0.281
Practical Training	1.015	0.038	1	0.845
Dedicated Training	0.864	1.607	1	0.205

a. Set to 1 because this parameter is redundant.

**Table 1.14**<br>Dependent variable: "Received help from other students or family members on assignments you were supposed to complete on your own."<br>Omnibus model test: Likelihood ratio chi2(8)=21.18; p=0.0067; n=1133.<br>Goodness of fit result: Ordinal HL=0.0554; PR p=0.1117; Lipsitz=0.0182.				
		**Hypothesis Test**		
**Parameter**	**OR**	**Wald Chi-Square**	**df**	**Sig.**
Threshold 1	0.188	7.014	1	0.008
Threshold 2	0.429	1.807	1	0.179
Threshold 3	3.478	3.902	1	0.048
Switzerland	0.888	0.691	1	0.406
Portugal	1.125	0.464	1	0.496
Ireland	1.277	1.690	1	0.194
Denmark (reference)	1[a]			
Other/don't want to tell	0.872	0.435	1	0.510
Female	1.070	0.314	1	0.575
Male (reference)	1[a]			
Age (in years)	0.983	0.267	1	0.605
Practical Training	1.115	2.139	1	0.144
Dedicated Training	0.728	7.827	1	0.005

a. Set to 1 because this parameter is redundant.

Table 1.15
Dependent variable: ”Copied shorter passages from other sources into your own text / research publication without marking them as quotes”.
Original model
Omnibus model test: Likelihood ratio chi2(8)=121.98; p<0.001; n=1099.
Goodness of fit result: Ordinal HL=0.2251; PR p=0.0422; Lipsitz=0.0367.
New model
Outcome variable recoded to a binary variable to obtain acceptable goodness of fit.
Omnibus model test: Likelihood ratio chi2(8)=85.15; p<0.001 ; n=1099.
Goodness of fit result: Hosmer-Lemeshow Goodness-of-fit test: chi2(8)=4.29; p=0.8305.

		Hypothesis Test		
**Parameter**	**OR**	**Wald Chi-Square**	**df**	**Sig.**
(Intercept)	0.963	0.003	1	0.958
Switzerland	0.840	1.175	1	0.278
Portugal	2.463	18.940	1	<0.001
Ireland	3.169	27.419	1	<0.001
Denmark (reference)	1[a]			
Other/don't want to tell	1.169	0.396	1	0.529
Female	0.843	1.486	1	0.223
Male (reference)	1[a]			
Age (in years)	1.014	0.131	1	0.717
Practical Training	0.909	1.292	1	0.256
Dedicated Training	0.884	0.873	1	0.350

a. Set to 1 because this parameter is redundant.

Bachelor level

Table 2.1
Dependent variable: "Copying an entire page stating a central point from an external source into your own text without quotation marks but including a reference".
Omnibus model test: Likelihood ratio chi2(8)=28.60; p<0.001; n=922.
Goodness of fit result: Hosmer-Lemeshow Goodness-of-fit test: chi2(8)=2.74; p=0.9495.

		Hypothesis Test		
**Parameter**	**OR**	**Wald Chi-Square**	**df**	**Sig.**
(Intercept)	18.461	28.820	1	<0.001
Switzerland	0.886	0.089	1	0.765
Portugal	0.374	8.470	1	0.004
Ireland	0.590	2.135	1	0.144
Denmark (reference)	1[a]			
Other/don't want to tell	0.636	0.993	1	0.319
Female	1.325	1.310	1	0.252
Male (reference)	1[a]			
Age (in years)	0.969	3.491	1	0.062
Practical Training	1.214	2.021	1	0.155
Dedicated Training	1.280	1.146	1	0.284

a. Set to 1 because this parameter is redundant.

Table 2.2
Dependent variable: "Copying one short paragraph stating a central point from an external source into your own text without quotation marks but including a reference."
Omnibus model test: Likelihood ratio chi2(8)=38.46; p<0.001; n=922.
Goodness of fit result: Hosmer-Lemeshow Goodness-of-fit test: chi2(8)=8.84; p=0.3557.

		Hypothesis Test		
**Parameter**	**OR**	**Wald Chi-Square**	**df**	**Sig.**
(Intercept)	7.205	18.109	1	<0.001
Switzerland	0.870	0.185	1	0.667
Portugal	0.379	12.588	1	<0.001
Ireland	0.507	5.716	1	0.017
Denmark (reference)	1[a]			
Other/don't want to tell	1.464	0.706	1	0.401
Female	1.427	3.269	1	0.071
Male (reference)	1[a]			
Age (in years)	0.981	1.653	1	0.199
Practical Training	1.219	3.226	1	0.072
Dedicated Training	1.327	2.341	1	0.126

a. Set to 1 because this parameter is redundant.

**Table 2.3**<br>Dependent variable: “Changing 10% of the words in a short paragraph stating a central point from an external source and using it in your own text with a reference.”<br>Omnibus model test: Likelihood ratio chi2(8)=5.27; p=0.7280; n=922.<br>Goodness of fit result: Hosmer-Lemeshow Goodness-of-fit test: chi2(8)=3.31; p=0.9136.				
		**Hypothesis Test**		
**Parameter**	**OR**	**Wald Chi-Square**	**df**	**Sig.**
(Intercept)	0.286	4.420	1	0.036
Switzerland	0.608	2.462	1	0.117
Portugal	0.714	1.403	1	0.236
Ireland	0.849	0.347	1	0.556
Denmark (reference)	1[a]			
Other/don't want to tell	0.703	0.396	1	0.529
Female	0.935	0.088	1	0.766
Male (reference)	1[a]			
Age (in years)	0.986	0.378	1	0.539
Practical Training	1.017	0.021	1	0.886
Dedicated Training	0.807	1.086	1	0.297

a. Set to 1 because this parameter is redundant.

**Table 2.4**<br>Dependent variable: “Copying a central point formulated in half a sentence from an external source without marking it with quotation marks but including a reference.”<br>Omnibus model test: Likelihood ratio chi2(8)=4.75; p=0.7839; n=922.<br>Goodness of fit result: Hosmer-Lemeshow Goodness-of-fit test: chi2(8)=15.12; p=0.0569.				
		**Hypothesis Test**		
**Parameter**	**OR**	**Wald Chi-Square**	**df**	**Sig.**
(Intercept)	0.214	6.787	1	0.009
Switzerland	0.915	0.087	1	0.768
Portugal	0.819	0.460	1	0.498
Ireland	0.852	0.305	1	0.580
Denmark (reference)	1[a]			
Other/don't want to tell	0.298	2.618	1	0.106
Female	0.816	0.847	1	0.358
Male (reference)	1[a]			
Age (in years)	0.991	0.182	1	0.670
Practical Training	1.015	0.016	1	0.900
Dedicated Training	1.044	0.043	1	0.835

a. Set to 1 because this parameter is redundant.

**Table 2.5**<br>Dependent variable: “Paying someone to write an assignment for you.”<br>Omnibus model test: Likelihood ratio chi2(8)=21.42; p=0.0061; n=922.<br>Goodness of fit result: Hosmer-Lemeshow Goodness-of-fit test: chi2(8)=7.20; p=0.5152.				
			**Hypothesis Test**	
**Parameter**	**OR**	**Wald Chi-Square**	**df**	**Sig.**
(Intercept)	32.750	14.474	1	<0.001
Switzerland	0.413	1.980	1	0.159
Portugal	0.172	9.755	1	0.002
Ireland	0.495	1.270	1	0.260
Denmark (reference)	1[a]			
Other/don't want to tell	1.792	0.566	1	0.452
Female	1.869	3.793	1	0.051
Male (reference)	1[a]			
Age (in years)	0.999	<0.001	1	0.986
Practical Training	1.171	0.708	1	0.400
Dedicated Training	0.945	0.032	1	0.858

a. Set to 1 because this parameter is redundant.

**Table 2.6**<br>Dependent variable: “Comparing answers to an individual assignment with other students before handing in the assignment.”<br><u>Original model</u><br>Omnibus model test: Likelihood ratio chi2(8)=18.56; p=0.0174 ; n=922.<br>Goodness of fit result: Hosmer-Lemeshow Goodness-of-fit test: chi2(8)=17.03; p=0.0298.<br><u>New model</u><br>Gender variable removed from analysis to obtain acceptable goodness of fit.<br>Omnibus model test: Likelihood ratio chi2(6)=17.58; p=0.0074 ; n=922.<br>Goodness of fit result: Hosmer-Lemeshow Goodness-of-fit test: chi2(8)=7.30; p=0.5046.				
			**Hypothesis Test**	
**Parameter**	**OR**	**Wald Chi-Square**	**df**	**Sig.**
(Intercept)	0.370	5.324	1	0.021
Switzerland	0.539	7.047	1	0.008
Portugal	0.622	4.471	1	0.034
Ireland	0.665	3.512	1	0.061
Denmark (reference)	1[a]			
Age (in years)	0.992	0.270	1	0.604
Practical Training	1.174	3.062	1	0.080
Dedicated Training	1.300	2.689	1	0.101

a. Set to 1 because this parameter is redundant.

**Table 2.7**<br>Dependent variable: "Handing in an assignment that you made with extensive help from another student or family member without mentioning the help you received."<br>Omnibus model test: Likelihood ratio chi2(8)=6.42; p=0.6006; n=922.<br>Goodness of fit result: Hosmer-Lemeshow Goodness-of-fit test: chi2(8)= 2.66; p= 0.9538.				
		**Hypothesis Test**		
**Parameter**	**OR**	**Wald Chi-Square**	**df**	**Sig.**
(Intercept)	0.209	11.788	1	0.001
Switzerland	1.004	<0.001	1	0.988
Portugal	1.073	0.089	1	0.766
Ireland	0.661	2.759	1	0.097
Denmark (reference)	1[a]			
Other/don't want to tell	0.834	0.166	1	0.684
Female	1.165	0.639	1	0.424
Male (reference)	1[a]			
Age (in years)	1.001	0.009	1	0.926
Practical Training	1.055	0.296	1	0.586
Dedicated Training	1.080	0.203	1	0.652

a. Set to 1 because this parameter is redundant.

**Table 2.8**<br>Dependent variable: "Let one member of a group do all the writing on a group project while the other members contribute to analysis and literature search."<br>Omnibus model test: Likelihood ratio chi2(8)=7.44; p=0.4902; n=922.<br>Goodness of fit result: Hosmer-Lemeshow Goodness-of-fit test: chi2(8)=14.15; p=0.0780.				
		**Hypothesis Test**		
**Parameter**	**OR**	**Wald Chi-Square**	**df**	**Sig.**
(Intercept)	0.333	6.830	1	0.009
Switzerland	1.132	0.289	1	0.591
Portugal	0.983	0.006	1	0.938
Ireland	0.834	0.623	1	0.430
Denmark (reference)	1[a]			
Other/don't want to tell	1.415	0.974	1	0.324
Female	0.750	2.718	1	0.099
Male (reference)	1[a]			
Age (in years)	1.002	0.027	1	0.870
Practical Training	1.022	0.054	1	0.816
Dedicated Training	1.025	0.024	1	0.878

a. Set to 1 because this parameter is redundant.

**Table 2.9**<br>Dependent variable: "Not mentioning in an assignment that you replaced a number of outliers in a data set with data points obtained through estimates based on the remaining data points."<br>Omnibus model test: Likelihood ratio chi2(8)= 45.88; p<0.001; n=744.<br>Goodness of fit result: Hosmer-Lemeshow Goodness-of-fit test: chi2(8)=3.90; p=0.8662.				
		**Hypothesis Test**		
**Parameter**	**OR**	**Wald Chi-Square**	**df**	**Sig.**
(Intercept)	3.071	2.602	1	0.107
Switzerland	1.614	1.428	1	0.232
Portugal	0.513	4.911	1	0.027
Ireland	0.574	3.253	1	0.071
Denmark (reference)	1[a]			
Other/don't want to tell	0.537	1.633	1	0.201
Female	0.711	2.070	1	0.150
Male (reference)	1[a]			
Age (in years)	1.025	0.885	1	0.347
Practical Training	1.454	8.534	1	0.003
Dedicated Training	1.382	2.466	1	0.116

a. Set to 1 because this parameter is redundant.

**Table 2.10**<br>Dependent variable: "Not mentioning in an assignment that you removed a number of deviating data points from a dataset when the cause of the deviation was unknown."<br>Omnibus model test: Likelihood ratio chi2(8)=31.49; p=0.001; n=774.<br>Goodness of fit result: Hosmer-Lemeshow Goodness-of-fit test: chi2(8)=9.96; p=0.2679.				
		**Hypothesis Test**		
**Parameter**	**OR**	**Wald Chi-Square**	**df**	**Sig.**
(Intercept)	2.373	3.618	1	0.057
Switzerland	1.492	2.549	1	0.110
Portugal	0.608	5.109	1	0.024
Ireland	0.766	1.471	1	0.225
Denmark (reference)	1[a]			
Other/don't want to tell	0.485	3.481	1	0.062
Female	0.706	3.896	1	0.048
Male (reference)	1[a]			
Age (in years)	0.998	0.017	1	0.896
Practical Training	1.084	0.776	1	0.378
Dedicated Training	1.212	1.516	1	0.218

a. Set to 1 because this parameter is redundant.

**Table 2.11**<br>Dependent variable: "Not mentioning in an assignment that you removed a number of deviating data points from a dataset when the cause of the deviation was known."<br>Omnibus model test: Likelihood ratio chi2(8)=5.68; p=0.6833; n=744.<br>Goodness of fit result: Hosmer-Lemeshow Goodness-of-fit test: chi2(8)=9.37; p=0.3120.				
		**Hypothesis Test**		
**Parameter**	**OR**	**Wald Chi-Square**	**df**	**Sig.**
(Intercept)	0.130	5.599	1	0.018
Switzerland	0.578	1.437	1	0.231
Portugal	1.432	0.959	1	0.327
Ireland	1.002	<0.001	1	0.996
Denmark (reference)	1[a]			
Other/don't want to tell	0.678	0.255	1	0.614
Female	0.913	0.102	1	0.749
Male (reference)	1[a]			
Age (in years)	0.980	0.380	1	0.538
Practical Training	1.073	0.213	1	0.645
Dedicated Training	1.106	0.147	1	0.702

a. Set to 1 because this parameter is redundant.

**Table 2.12**<br>Dependent variable: "Deleted or ignored deviating or unusual data based on a gut feeling that they were inaccurate or unreliable."<br>Omnibus model test: Likelihood ratio chi2(8)=10.49; p=0.2325; n=665.<br>Goodness of fit result: Ordinal HL=0.2270; PR p=0.1010; Lipsitz=0.1965.				
		**Hypothesis Test**		
**Parameter**	**OR**	**Wald Chi-Square**	**df**	**Sig.**
Threshold 1	2.065	2.052	1	0.152
Threshold 2	5.874	11.941	1	0.001
Threshold 3	115.188	49.890	1	<0.001
Switzerland	0.917	0.102	1	0.750
Portugal	1.733	4.961	1	0.026
Ireland	1.337	1.409	1	0.235
Denmark (reference)	1[a]			
Other/don't want to tell	1.162	0.109	1	0.742
Female	0.852	0.768	1	0.381
Male (reference)	1[a]			
Age (in years)	0.986	0.589	1	0.443
Practical Training	1.004	0.001	1	0.970
Dedicated Training	1.049	0.077	1	0.781

a. Set to 1 because this parameter is redundant.

Table 2.13
Dependent variable: "Performed a misleading or dubious interpretation or statistical analysis of data, texts, works of art, or interviews to achieve results that the teacher would accept / a publishable."
Original model
Omnibus model test: Likelihood ratio chi2(8)=23.06 ; p=0.0033; n=698.
Goodness of fit result: Ordinal HL= missing; PR p=missing; Lipsitz=0.1218.
New model
Outcome variable recoded to a binary variable to obtain acceptable goodness of fit.
Omnibus model test: Likelihood ratio chi2(8)=21.92; p=0.0051; n=698.
Goodness of fit result: Hosmer-Lemeshow Goodness-of-fit test: chi2(8)=10.67; p=0.2212.

		**Hypothesis Test**		
**Parameter**	**OR**	**Wald Chi-Square**	**df**	**Sig.**
(Intercept)	0.632	0.346	1	0.556
Switzerland	0.456	4.661	1	0.031
Portugal	1.527	2.137	1	0.144
Ireland	1.295	0.827	1	0.363
Denmark (reference)	1[a]			
Other/don't want to tell	0.855	0.089	1	0.766
Female	0.868	0.415	1	0.519
Male (reference)	1[a]			
Age (in years)	0.941	3.653	1	0.056
Practical Training	1.125	0.943	1	0.332
Dedicated Training	1.261	1.313	1	0.252

a. Set to 1 because this parameter is redundant.

**Table 2.14**<br>Dependent variable: "Added students as co-authors of group assignments, even though they did not contribute."<br>Omnibus model test: Likelihood ratio chi2(8)=125.80; p<0.001; n=849.<br>Goodness of fit result: Ordinal HL=0.2510; PR p=0.5346; Lipsitz=0.1686.				
		**Hypothesis Test**		
**Parameter**	**OR**	**Wald Chi-Square**	**df**	**Sig.**
Threshold 1	2.046	2.905	1	0.088
Threshold 2	4.975	14.415	1	<0.001
Threshold 3	34.308	63.150	1	<0.001
Switzerland	0.387	18.249	1	<0.001
Portugal	3.469	41.817	1	<0.001
Ireland	0.696	3.384	1	0.066
Denmark (reference)	1[a]			
Other/don't want to tell	0.877	0.120	1	0.729
Female	1.243	1.963	1	0.161
Male (reference)	1[a]			
Age (in years)	0.997	0.047	1	0.828
Practical Training	1.171	3.751	1	0.053
Dedicated Training	1.279	3.094	1	0.079

a. Set to 1 because this parameter is redundant.

**Table 2.15**<br>Dependent variable: "Received help from other students or family members on assignments you were supposed to complete on your own."<br>Omnibus model test: Likelihood ratio chi2(8)=34.56; p<0.001; n=870.<br>Goodness of fit result: Ordinal HL=0.2592; PR p=0.1745; Lipsitz=0.8322.				
		**Hypothesis Test**		
**Parameter**	**OR**	**Wald Chi-Square**	**df**	**Sig.**
Threshold 1	0.626	1.612	1	0.204
Threshold 2	1.305	0.520	1	0.471
Threshold 3	13.993	46.002	1	<0.001
Switzerland	1.507	4.362	1	0.037
Portugal	2.425	22.046	1	<0.001
Ireland	1.809	10.091	1	0.001
Denmark (reference)	1[a]			
Other/don't want to tell	0.835	0.277	1	0.599
Female	0.841	1.414	1	0.234
Male (reference)	1[a]			
Age (in years)	0.976	3.386	1	0.066
Practical Training	1.020	0.065	1	0.799
Dedicated Training	0.920	0.432	1	0.511

a. Set to 1 because this parameter is redundant.

**Table 2.16**<br>Dependent variable: "Copied shorter passages from other sources into your own text / research publication without marking them as quotes."<br>Omnibus model test: Likelihood ratio chi2(8)=74.61; p<0.001; n=855.<br>Goodness of fit result: Ordinal HL=0.5535; PR p=0.8782; Lipsitz=0.8579.				
		**Hypothesis Test**		
**Parameter**	**OR**	**Wald Chi-Square**	**df**	**Sig.**
Threshold 1	2.715	3.741	1	0.053
Threshold 2	5.837	11.534	1	0.001
Threshold 3	50.772	47.266	1	<0.001
Switzerland	0.940	0.037	1	0.848
Portugal	3.619	25.194	1	<0.001
Ireland	2.187	8.735	1	0.003
Denmark (reference)	1[a]			
Other/don't want to tell	0.495	2.133	1	0.144
Female	0.835	0.963	1	0.327
Male (reference)	1[a]			
Age (in years)	0.982	0.893	1	0.345
Practical Training	0.888	1.355	1	0.244
Dedicated Training	0.703	4.314	1	0.038

a. Set to 1 because this parameter is redundant.

**Table 2.17**<br>Dependent variable: "Kept inadequate records of parts of your work that should be documented."<br>Original model<br>Omnibus model test: Likelihood ratio chi2(8)=16.87; p=0.0314; n=609.<br>Goodness of fit result: Ordinal HL=0.8251; PR p=0.0666; Lipsitz=0.0214.<br>New model<br>Outcome variable recoded to a binary variable and gender variable removed to obtain acceptable goodness of fit.<br>Omnibus model test: Likelihood ratio chi2(6)=9.57; p=0.1439; n=609.<br>Goodness of fit result: Hosmer-Lemeshow Goodness-of-fit test: chi2(8)=7.00; p=0.5361.				
		**Hypothesis Test**		
**Parameter**	**OR**	**Wald Chi-Square**	**df**	**Sig.**
(Intercept)	0.288	6,080	1	0,014
Switzerland	0.725	1,301	1	0,254
Portugal	0.952	0,032	1	0,857
Ireland	1.240	0,729	1	0,393
Denmark (reference)	1[a]			
Age (in years)	0.994	0,095	1	0,758
Practical Training	1.158	1,789	1	0,181
Dedicated Training	1.312	2,030	1	0,154

a. Set to 1 because this parameter is redundant.

PhD level

Table 3.1
Dependent variable: “Copying an entire page stating a central point from an external source into your own text without quotation marks but including a reference.”
Omnibus model test: Likelihood ratio chi2(8)=18.47; p=0.0180; n=1115.
Goodness of fit result: Hosmer-Lemeshow Goodness-of-fit test: chi2(8)=9.25; p=0.3218.

			Hypothesis Test		
**Parameter**	**OR**	**Std. Error**	**Wald Chi-Square**	**df**	**Sig.**
(Intercept)	30.564	0.6758	25.607	1	<0.001
Switzerland	0.482	0.3888	3.517	1	0.061
Portugal	0.443	0.3799	4.598	1	0.032
Ireland	0.722	0.3883	0.704	1	0.402
Denmark (reference)	1[a]				
Other/don't want to tell	0.229	0.4141	12.671	1	<0.001
Female	0.930	0.2998	0.059	1	0.809
Male (reference)	1[a]				
Age (in years)	1.009	0.0168	0.254	1	0.614
Practical Training	0.999	0.1440	<0.001	1	0.996
Dedicated Training	0.732	0.2660	1.380	1	0.240

a. Set to 1 because this parameter is redundant.

Table 3.2
Dependent variable: “Copying one short paragraph stating a central point from an external source into your own text without quotation marks but including a reference.”
<u>Original</u>
Omnibus model test: Likelihood ratio chi2(8)=25.73; p=0.0012; n=1115.
Goodness of fit result: Hosmer-Lemeshow Goodness-of-fit test: chi2(8)=15.54; p=0.0495.
<u>New model</u>
Age variable removed from analysis to obtain acceptable goodness of fit.
Omnibus model test: Likelihood ratio chi2(8)=24.07; p=0.0011; n=1115.
Goodness of fit result: Hosmer-Lemeshow Goodness-of-fit test: chi2(8)=6.82; p=0.5564.

			Hypothesis Test		
**Parameter**	**OR**	**Std. Error**	**Wald Chi-Square**	**df**	**Sig.**
(Intercept)	10.571	0.3178	55.044	1	<0.001
Switzerland	0.764	0.3251	0.686	1	0.407
Portugal	0.618	0.2944	2.682	1	0.102
Ireland	0.807	0.3069	0.486	1	0.486
Denmark (reference)	1[a]				
Other/don't want to tell	0.330	0.3392	10.690	1	0.001
Female	1.526	0.2353	3.229	1	0.072
Male (reference)	1[a]				
Practical Training	1.017	0.1187	0.021	1	0.886
Dedicated Training	1.162	0.2187	0.470	1	0.493

a. Set to 1 because this parameter is redundant.

Table 3.3
Dependent variable: “Changing 10% of the words in a short paragraph stating a central point from an external source and using it in your own text with a reference.”
Omnibus model test: Likelihood ratio chi2(8)=10.18; p=0.2525; n=1115.
Goodness of fit result: Hosmer-Lemeshow Goodness-of-fit test: chi2(8)=5.59; p=0.6933.

			Hypothesis Test		
**Parameter**	**OR**	**Std. Error**	**Wald Chi-Square**	**df**	**Sig.**
(Intercept)	0.186	0.4445	14.301	1	<0.001
Switzerland	1.279	0.2407	1.045	1	0.307
Portugal	1.245	0.2423	0.816	1	0.366
Ireland	0.755	0.2524	1.236	1	0.266
Denmark (reference)	1[a]				
Other/don't want to tell	1.468	0.3665	1.097	1	0.295
Female	1.242	0.1854	1.371	1	0.242
Male (reference)	1[a]				
Age (in years)	0.986	0.0116	1.393	1	0.238
Practical Training	1.119	0.0930	1.456	1	0.228
Dedicated Training	0.973	0.1763	0.025	1	0.875

a. Set to 1 because this parameter is redundant.

Table 3.4
Dependent variable: “Copying a central point formulated in half a sentence from an external source without marking it with quotation marks but including a reference.”
Omnibus model test: Likelihood ratio chi2(8)=21.58; p=0.0058; n=1115.
Goodness of fit result: Hosmer-Lemeshow Goodness-of-fit test: chi2(8)=6.21; p=0.6235.

			Hypothesis Test		
**Parameter**	**OR**	**Std. Error**	**Wald Chi-Square**	**df**	**Sig.**
(Intercept)	0.473	0.4929	2.306	1	0.129
Switzerland	1.024	0.2395	0.010	1	0.921
Portugal	0.799	0.2587	0.751	1	0.386
Ireland	0.551	0.2583	5.329	1	0.021
Denmark (reference)	1[a]				
Other/don't want to tell	1.773	0.3596	2.536	1	0.111
Female	1.193	0.1891	0.869	1	0.351
Male (reference)	1[a]				
Age (in years)	0.962	0.0139	7.824	1	0.005
Practical Training	1.045	0.0940	0.217	1	0.641
Dedicated Training	1.140	0.1831	0.514	1	0.473

a. Set to 1 because this parameter is redundant.

**Table 3.5**<br>Dependent variable: “Not mentioning in an assignment that you replaced a number of outliers in a data set with data points obtained through estimates based on the remaining data points.”<br>Omnibus model test: Likelihood ratio chi2(8)=75.68; p<0.001; n=996.<br>Goodness of fit result: Hosmer-Lemeshow Goodness-of-fit test: chi2(8)=6.29; p=0.6147.					
			**Hypothesis Test**		
**Parameter**	**OR**	**Std. Error**	**Wald Chi-Square**	**df**	**Sig.**
(Intercept)	7.484	0.5769	12.172	1	<0.001
Switzerland	0.608	0.4105	1.466	1	0.226
Portugal	0.136	0.3283	36.897	1	<0.001
Ireland	0.461	0.3738	4.300	1	0.038
Denmark (reference)	1[a]				
Other/don't want to tell	0.421	0.4051	4.553	1	0.033
Female	1.027	0.2469	0.012	1	0.914
Male (reference)	1[a]				
Age (in years)	1.028	0.0139	3.908	1	0.048
Practical Training	0.995	0.1269	0.002	1	0.966
Dedicated Training	1.681	0.2293	5.135	1	0.023

a. Set to 1 because this parameter is redundant.

**Table 3.6**<br>Dependent variable: “Not mentioning in an assignment that you removed a number of deviating data points from a dataset when the cause of the deviation was unknown.”<br>Omnibus model test: Likelihood ratio chi2(8)=59.88; p<0.001; n=996.<br>Goodness of fit result: Hosmer-Lemeshow Goodness-of-fit test: chi2(8)=4.90; p=0.7680.					
			**Hypothesis Test**		
**Parameter**	**OR**	**Std. Error**	**Wald Chi-Square**	**df**	**Sig.**
(Intercept)	7.883	0.3995	26.709	1	<0.001
Switzerland	0.885	0.2627	0.216	1	0.642
Portugal	0.373	0.2216	19.789	1	<0.001
Ireland	0.880	0.2438	0.276	1	0.599
Denmark (reference)	1[a]				
Other/don't want to tell	0.491	0.3430	4.312	1	0.038
Female	0.775	0.1806	1.997	1	0.158
Male (reference)	1[a]				
Age (in years)	0.989	0.0095	1.465	1	0.226
Practical Training	0.925	0.0912	0.741	1	0.389
Dedicated Training	1.725	0.1700	10.297	1	0.001

a. Set to 1 because this parameter is redundant.

**Table 3.7**<br>Dependent variable: "Not mentioning in an assignment that you removed a number of deviating data points from a dataset when the cause of the deviation was known."<br>Omnibus model test: Likelihood ratio chi2(8)=7.19; p=0.5166; n=996.<br>Goodness of fit result: Hosmer-Lemeshow Goodness-of-fit test: chi2(8)=7.35; p=0.4997.					
			**Hypothesis Test**		
**Parameter**	**OR**	**Std. Error**	**Wald Chi-Square**	**df**	**Sig.**
(Intercept)	0.076	0.5437	22.492	1	<0.001
Switzerland	0.856	0.3373	0.213	1	0.644
Portugal	1.078	0.3062	0.060	1	0.807
Ireland	0.994	0.3023	<0.001	1	0.985
Denmark (reference)	1[a]				
Other/don't want to tell	1.416	0.4797	0.526	1	0.468
Female	1.262	0.2384	0.953	1	0.329
Male (reference)	1[a]				
Age (in years)	1.002	0.0133	0.018	1	0.894
Practical Training	1.267	0.1193	3.933	1	0.047
Dedicated Training	0.694	0.2275	2.573	1	0.109

a. Set to 1 because this parameter is redundant.

**Table 3.8**<br>Dependent variable: "Deleted or ignored deviating or unusual data based on a gut feeling that they were inaccurate or unreliable"<br>Omnibus model test: Likelihood ratio chi2(8)=24.23; p=0.0021; n=912.<br>Goodness of fit result: Ordinal HL=0.5741; PR p=0.2512; Lipsitz=0.8091.					
			**Hypothesis Test**		
**Parameter**	**OR**	**Std. Error**	**Wald Chi-Square**	**df**	**Sig.**
Threshold 1	2.264	0.4851	2.837	1	0.092
Threshold 2	6.613	0.4957	14.522	1	<0.001
Threshold 3	84.861	0.6877	41.701	1	<0.001
Switzerland	1.425	0.3008	1.389	1	0.239
Portugal	1.746	0.2802	3.953	1	0.047
Ireland	2.191	0.2595	9.140	1	0.003
Denmark (reference)	1[a]				
Other/don't want to tell	0.473	0.5548	1.825	1	0.177
Female	0.821	0.2018	0.953	1	0.329
Male (reference)	1[a]				
Age (in years)	0.980	0.0124	2.645	1	0.104
Practical Training	0.802	0.1080	4.166	1	0.041
Dedicated Training	0.646	0.1994	4.804	1	0.028

a. Set to 1 because this parameter is redundant.

Table 3.9
Dependent variable: "Performed a misleading or dubious interpretation or statistical analysis of data, texts, works of art, or interviews to achieve results that the teacher would accept / a publishable"
Original model
Omnibus model test: Likelihood ratio chi2(8)=13.80; p=0.0872; n=910.
Goodness of fit result: Ordinal HL=0.6123; PR p=missing; Lipsitz=0.0735.
New model
Gender variable removed from analysis to obtain acceptable goodness of fit.
Omnibus model test: Likelihood ratio chi2(6)=9.36; p=0.1543; n=910.
Goodness of fit result: Ordinal HL=0.8339; PR p=0.9426; Lipsitz=0.0923.

			Hypothesis Test		
**Parameter**	**OR**	**Std. Error**	**Wald Chi-Square**	**df**	**Sig.**
Threshold 1	8.619	0.9318	5.344	1	0.021
Threshold 2	27.530	0.9636	11.837	1	0.001
Threshold 3	102.142	1.0821	18.279	1	<0.001
Switzerland	0.606	0.6566	0.580	1	0.446
Portugal	1.264	0.5260	0.198	1	0.656
Ireland	1.644	0.4174	1.419	1	0.234
Denmark (reference)	1[a]				
Age (in years)	0.955	0.0265	2.998	1	0.083
Practical Training	0.881	0.1896	0.450	1	0.502
Dedicated Training	1.645	0.3716	1.792	1	0.181

a. Set to 1 because this parameter is redundant.

**Table 3.10**<br>Dependent variable: "Copied shorter passages from other sources into your own text / research publication without marking them as quotes."<br>Omnibus model test: Likelihood ratio chi2(8)=14.90; p=0.0611; n=1046.<br>Goodness of fit result: Ordinal HL=0.6833; PR p=0.7169; Lipsitz=0.7196.					
			**Hypothesis Test**		
**Parameter**	**OR**	**Std. Error**	**Wald Chi-Square**	**df**	**Sig.**
Threshold 1	12.466	0.5712	19.507	1	<0.001
Threshold 2	27.601	0.5832	32.362	1	<0.001
Threshold 3	272.246	0.7503	55.840	1	<0.001
Switzerland	1.043	0.3953	0.011	1	0.915
Portugal	1.886	0.3300	3.696	1	0.055
Ireland	1.799	0.3030	3.753	1	0.053
Denmark (reference)	1[a]				
Other/don't want to tell	1.088	0.5128	0.027	1	0.869
Female	0.943	0.2472	0.055	1	0.814
Male (reference)	1[a]				
Age (in years)	0.992	0.0141	0.292	1	0.589
Practical Training	0.752	0.1301	4.800	1	0.028
Dedicated Training	1.605	0.2390	3.919	1	0.048

a. Set to zero because this parameter is redundant.

**Table 3.11**<br>Dependent variable: "Kept inadequate records of parts of your work that should be documented"<br><u>Original model</u><br>Omnibus model test: Likelihood ratio chi2(8)=37.79; p<0.001; n=859.<br>Goodness of fit result: Ordinal HL=0.0226; PR p=0.0018; Lipsitz=0.7790.<br><u>New model</u><br>Outcome variable recoded to a binary variable to obtain acceptable goodness of fit.<br>Omnibus model test: Likelihood ratio chi2(8)=35.25; p<0.001; n=859.<br>Goodness of fit result: Hosmer-Lemeshow Goodness-of-fit test: chi2(8)=4.57; p=0.8026.					
			**Hypothesis Test**		
**Parameter**	**OR**	**Std. Error**	**Wald Chi-Square**	**df**	**Sig.**
(Intercept)	0.679	0.4520	0.734	1	0.392
Switzerland	0.676	0.2762	2.017	1	0.156
Portugal	0.509	0.2885	5.472	1	0.019
Ireland	1.733	0.2075	7.017	1	0.008
Denmark (reference)	1[a]				
Other/don't want to tell	0.719	0.4750	0.484	1	0.487
Female	1.065	0.1820	0.119	1	0.730
Male (reference)	1[a]				
Age (in years)	0.967	0.0120	7.840	1	0.005
Practical Training	1.094	0.0919	0.958	1	0.328
Dedicated Training	0.959	0.1820	0.054	1	0.816

a. Set to zero because this parameter is redundant.
