# Supplementary material for: Academic integrity across educational levels: Exploring students’ engagement with grey-zone and non-compliant practices in four European countries
Source: PLoS One. 2026 Mar 4;21(3):e0342227. doi: 10.1371/journal.pone.0342227 (PMC12959713; doi:10.1371/journal.pone.0342227)
Supplement: S4 File — (PDF) [file pone.0342227.s004.pdf]

Supporting information S4: Marginal effects from regression analysis.

Note: Here the marginal effects from the two training variables ('dedicated training' and 'practical training') are reported for the models where at least one of them were statistically significant ($p<0.05$). Marginal effects from each educational level (upper secondary, bachelor and PhD) are reported separately.

The variable 'dedicated training' is a composite score (range 0-2) based on how many of two possible trainings the respondent has received: 'One or more dedicated courses or lectures' and 'One or more dedicated e sessions' (see Table 1 in S3 for descriptive statistics).

The variable 'practical training' is a composite score (range 0-3) based on how many of three possible training types the respondent has received: 'Feedback on written work or assignments in another course', 'In courses not dedicated exclusively to academic integrity' and 'Through discussions with teachers/seinor staff outside regular courses' (see Table 2 in S3 for descriptive statistics)

Marginal effects are reported using Stata's postestimation command *margins* where the *dydx* option was used. In the tables below, for all measures of rule conception, there is one dy/dx value. This value indicate the change in proportion when there is a 1-unit change in the predictor variable that is being reported about. All other other variables were set to the mean of the study sample (using Stata's *margins, atmeans* option).

For all measures of questionable behavior, there are four dy/dx values; one for each response option of the outcome variable (1=never to 4=yes, many times had four ord). The dy/dx values indicates the change in proportion for each response when there is a 1-unit change in the predictor variable that is being reported about. All other other variables were set to the mean of the study sample (using Stata's *margins, atmeans* option).

Upper secondary level participants

Table 1.1
Rule conception: Copying an entire page stating a central point from an external source into your own text without quotation marks but including a reference.
Predictor variable: Practical training (p<0.001).

	Delta method				
	dy/dx	std. err.	z	p>ızı	95% conf. interval
Practical training	0.0853963	0.0159495	5.35	0.000	0.0541359 – 0.1166567

Table 1.2
Rule conception: Copying a central point formulated in half a sentence from an external source without marking it with quotation marks but including a reference.
Predictor variable: Practical training (p<0.001).

	Delta method				
	dy/dx	std. err.	z	p>ızı	95% conf. interval
Practical training	0.0705259	0.0191119	3.69	0.000	0.0330672 – 0.1079846

Table 1.3
Rule conception: Paying someone to write an assignment for you.
Predictor variable: Practical training (p<0.001)

	Delta method				
	dy/dx	std. err.	z	p>ızı	95% conf. interval
Practical training	0.0918552	0.0146244	6.28	0.000	0.0631918 – 0.1205185

Table 1.4
Rule conception: Paying someone to write an assignment for you.
Predictor variable: Dedicated training (p<0.037).

	Delta method				
	dy/dx	std. err.	z	p>ızı	95% conf. interval
Dedicated training	-0.0428396	0.0204608	-2.09	0.036	-0.082942 – -0.0027373

Table 1.5
Rule conception: Not mentioning in an assignment that you replaced a number of outliers in a data set with data points obtained through estimates based on the remaining data points.
Predictor variable: Practical training (p<0.001).

	Delta method				
	dy/dx	std. err.	z	p>ızı	95% conf. interval
Practical training	0.0762115	0.0193363	3.94	0.000	0.0383129 – 0.11411

**Table 1.6**<br>Rule conception: Not mentioning in an assignment that you removed a number of deviating data points from a data set when the cause of the deviation was unknown.<br>Predictor variable: Practical training (p<0.001).					
	Delta method				
	dy/dx	std. err.	z	p>ızı	95% conf. interval
Practical training	0.089575	0.0190748	4.70	0.000	.0521892 – .1269609

**Table 1.7**<br>Questionable behavior: Deleted or ignored deviating or unusual data based on a gut feeling that they were inaccurate or unreliable.<br>Predictor variable: Practical training (p=0.005).					
	Delta method				
	dy/dx	std. err.	z	p>ızı	95% conf. interval
Practical training	0.0604353	0.0213069	2.84	0.005	0.0186746 – 0.102196

**Table 1.8**<br>Questionable behavior: Received help from other students or family members on assignments you were supposed to complete on your own.<br>Predictor variable: Dedicated training (p=0.005).					
	Delta method				
Dedicated training	dy/dx	std. err.	z	p>ızı	95% conf. interval
Never	0.050364	0.0180226	2.79	0.005	0.0150404 – 0.0856876
Yes, once	0.0228253	0.0084199	2.71	0.007	0.0063225 – 0.039328
Yes, a few times	-0.0262928	0.0099472	-2.64	0.008	-0.045789 – -0.0067966
Yes, many times	-0.0468964	0.0168015	-2.79	0.005	-0.0798268 – -0.013966

Bachelor level participants

**Table 2.1**<br>Rule conception: Not mentioning in an assignment that you replaced a number of outliers in a data set with data points obtained through estimates based on the remaining data points.<br>Predictor variable: Practical training (p=0.003).					
	Delta method				
	dy/dx	std. err.	z	p>ız|	95% conf. interval
Practical training	0.0447473	0.0150216	2.98	0.003	0.0153056 – 0.0741891

**Table 2.2**<br>Questionable behavior: Copied shorter passages from other sources into your own text / research publication without marking them as quotes.<br>Predictor variable: Dedicated training (p=0.038).					
	Delta method				
Dedicated training	dy/dx	std. err.	z	p>ız|	95% conf. interval
Never	0.0576744	0.0276884	2.08	0.037	0.0034063 – 0.1119426
Yes, once	-0.0237848	0.0116641	-2.04	0.041	-0.046646 – -0.0009236
Yes, a few times	-0.0291281	0.0141152	-2.06	0.039	-0.0567934 – -0.0014627
Yes, many times	-0.0047616	0.0025525	-1.87	0.062	-0.0097644 – 0.0002412

PhD level participants

**Table 3.1**<br>Rule conception: Not mentioning in an assignment that you replaced a number of outliers in a data set with data points obtained through estimates based on the remaining data points.<br>Predictor variable: Dedicated training (p=0.023).					
	Delta method				
	dy/dx	std. err.	z	p>ızı	95% conf. interval
Practical training	0.0340913	0.0149559	2.28	0.023	0.0047783 – 0.0634044

**Table 3.2**<br>Rule conception: Not mentioning in an assignment that you removed a number of deviating data points from a dataset when the cause of the deviation was unknown.<br>Predictor variable: Dedicated training (p=0.001).					
	Delta method				
	dy/dx	std. err.	z	p>ızı	95% conf. interval
Dedicated training	0.0794388	0.0244935	3.24	0.001	0.0314325 – 0.1274452

**Table 3.3**<br>Questionable behavior: Deleted or ignored deviating or unusual data based on a gut feeling that they were inaccurate or unreliable.<br>Predictor variable: Practical training (p=0.041).					
	Delta method				
Practical training	dy/dx	std. err.	z	p>ızı	95% conf. interval
Never	0.0245502	0.0118975	2.06	0.039	0.0012316 – 0.0478688
Yes, once	-0.0145353	0.0071604	-2.03	0.042	-0.0285694 – -0.0005012
Yes, a few times	-0.009161	0.0045451	-2.02	0.044	-0.0180693 – -0.0002527
Yes, many times	-0.000854	0.0005857	-1.46	0.145	-0.002002 – 0.0002941

**Table 3.4**<br>Questionable behavior: Deleted or ignored deviating or unusual data based on a gut feeling that they were inaccurate or unreliable.<br>Predictor variable: Dedicated training (p=0.028).					
	Delta method				
Dedicated training	dy/dx	std. err.	z	p>ızı	95% conf. interval
Never	0.0486813	0.0220026	2.21	0.027	0.0055571 – 0.0918056
Yes, once	-0.0288224	0.0132787	-2.17	0.030	-0.0548481 – -0.0027967
Yes, a few times	-0.0181655	0.0084309	-2.15	0.031	-0.0346897 – -.0016413
Yes, many times	-0.0016934	0.0011223	-1.51	0.131	-0.003893 – .0005063
